# Practical Approaches for Detecting Selection in Microbial Genomes

**DOI:** 10.1371/journal.pcbi.1004739

**Published:** 2016-02-11

**Authors:** Jessica Hedge, Daniel J. Wilson

**Affiliations:** 1 Nuffield Department of Medicine, University of Oxford, John Radcliffe Hospital, Oxford, United Kingdom; 2 Wellcome Trust Centre for Human Genetics, Oxford, United Kingdom; Ontario Institute for Cancer Research, CANADA

## Abstract

Microbial genome evolution is shaped by a variety of selective pressures. Understanding how these processes occur can help to address important problems in microbiology by explaining observed differences in phenotypes, including virulence and resistance to antibiotics. Greater access to whole-genome sequencing provides microbiologists with the opportunity to perform large-scale analyses of selection in novel settings, such as within individual hosts. This tutorial aims to guide researchers through the fundamentals underpinning popular methods for measuring selection in pathogens. These methods are transferable to a wide variety of organisms, and the exercises provided are designed for researchers with any level of programming experience.

This is part of the *PLOS Computational Biology* Education collection.

## Introduction

Whole-genome sequencing (WGS) of microbial samples is now affordable and fast, which has enabled its widespread use in both research and clinical practice [1–3]. Analysis of the genetic variation within WGS data can help characterize the selective pressures acting on microbial populations [4,5] and provide novel insight into infectious disease transmission [6], the emergence of antibiotic resistance [7,8], and the population dynamics of bacterial epidemics [9,10]. Selection acts on both existing and novel mutations that arise in individuals within a population by removing those mutations detrimental to the fitness of the individual and favoring those that are beneficial. This process can leave a signature across the genome sequences within the population that can reveal which regions are under functional constraint [5,11] or that are rapidly adapting to changes in the environment [12].

This tutorial aims to provide microbiologists possessing limited experience in population genetics analyses with (i) training in statistical methods for detecting selection, (ii) familiarity with the underlying theory, and (iii) an awareness of the assumptions and limitations of these methods. A wide variety of approaches are available to address many questions regarding microbial evolution, and deciding which to take will depend on numerous factors. These include the evolutionary processes acting on the sequences, level of genetic variation present within the data, and computational resources available to the researcher. Here, we provide one approach to performing a basic population genetics analysis of evolution and selection in non-recombining microbial populations and a supplementary exercise demonstrating how these methods can be applied to bacterial WGS data (S1 File). Further examples of where these methods have been employed to address a variety of evolutionary questions in microbial genomics are described in S1 Table.

These methods are not robust to homologous recombination and are therefore applicable when it is absent. It is also assumed that short-read sequence data have been aligned to a reference sequence and single nucleotide variants have been detected. The preceding steps in a typical bioinformatics pipeline are described in a number of recent reviews [13,14]. This guide is based on a workshop included as part of a course entitled “Genotype to Phenotype Mapping of Complex Traits” at the European Bioinformatics Institute at the Wellcome Trust Genome Campus (United Kingdom) in July 2014.

## Step 1: Construction of a Phylogenetic Tree

**Fig 1:**
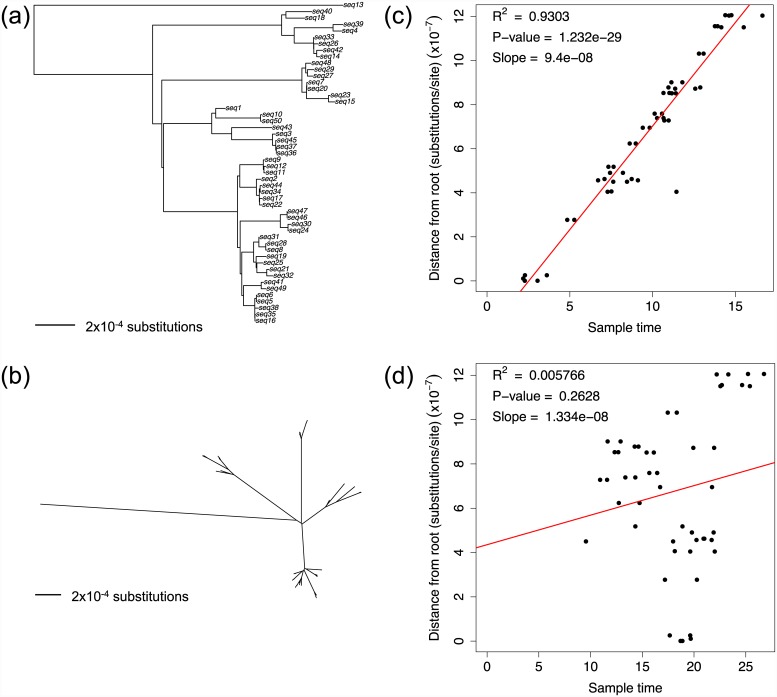
Phylogenetic tree reconstruction and evolutionary rate estimation. A phylogenetic tree comprises a collection of branches that connect sampled sequences at the tips (called taxa) with the most recent common ancestor of the sample. The point where each pair of branches join together is called a node. The lengths of these branches represent the evolutionary distance between sequences at either end, usually measured in numbers of substitutions per site, which can be calculated using the scale bar. The length of the vertical branches and rotation of branches around each node are arbitrary. The tree can be rooted using a divergent sequence (called an outgroup) (a), in which case the direction of substitutions can be inferred and each node represents the common ancestor of all descendent nodes and taxa. The node furthest from the tips is called the root. The tree can also be left unrooted and displayed radially (b) (tip labels have been omitted for visual clarity). Assuming the phylogeny has been rooted correctly, linear regression analysis can be used to test for a signal of a molecular clock by plotting the sampling time of each sequence against its evolutionary distance from the root of the tree. If the test is significant (c), the slope of the regression line (red) can provide an estimate of the evolutionary rate. The lack of any temporal signal (d) may occur if insufficient time has passed for substitutions to accumulate or if the molecular clock has been violated (for example, due to selection, recombination, or hypermutation).

Phylogenetic tree methods attempt to reconstruct the evolutionary relationships between a set of sampled sequences (Fig 1a and 1b). Construction of a phylogenetic tree can help to visualize the genetic relatedness between samples, infer the order of branching events, and provide one way to estimate important evolutionary parameters (such as the evolutionary rate, in Step 2). If sequences are sampled from multiple hosts, the phylogeny can also help to infer the transmission history during an epidemic [15–17]. Further details and examples of phylogenetic tree construction and interpretation can be found in several excellent resources (e.g., [18–20]).

Tree-building methods broadly fall into two categories. Distance-based methods use a clustering algorithm to sequentially group clusters of sequences, which makes them relatively fast. These include neighbor-joining (NJ) and un-weighted pair group method with arithmetic mean (UPGMA). However, neither method explicitly models back mutations or multiple hits (successive substitutions at a single site). Character-based methods evaluate a set of plausible trees based on certain criteria. This makes these methods slower, but information regarding the evolutionary history encoded by the characters is retained. These methods include maximum parsimony (MP), maximum likelihood (ML), and Bayesian methods. MP attempts to minimize the number of character changes across the tree. However, this can often underestimate the length of branches. ML and Bayesian methods are more popular, since they allow for specification of a probabilistic model of sequence evolution. These methods enable arbitrarily complex models of sequence evolution, but in the within-host context there may be limited data for reliable inference of highly-parameterized models, and simple models such as Jukes-Cantor may suffice. ML searches for the single tree with the greatest likelihood given the model, while Bayesian methods capture uncertainty in the tree by providing a distribution of trees that are likely given the data and explicit prior beliefs. Many phylogenetic analyses assume that sequences have evolved independently and under a constant evolutionary rate. However, in the presence of selection, convergent evolution may occur, in which the same substitution arises on different branches, which can cause some sequences on the tree to be inferred as more closely related than they truly are.

A variety of programs are available for performing phylogenetic analyses of microbial populations. Several methods, including distance-based methods, can easily be carried out using the ape library in R. PhyML and RAxML are popular programs for ML analysis of small and larger datasets, respectively, while the Bayesian phylogenetics software BEAST is commonly employed for estimating time-calibrated trees [21–24].

Analysis of the observed number of substitutions between sequences alone is usually not sufficient to describe the underlying evolutionary process for a set of sequences. Principled statistical inference of phylogenetic trees requires specification of a sequence substitution model, describing the base frequencies (*f*_i_) and rate of change from allele *i* (rows) to allele *j* (columns) (*r*_ij_) via entry *Q*_ij_ of a substitution rate matrix, *Q*. For example, for the general time-reversible nucleotide substitution model (GTR):

$$Q=\left[\begin{array}{ccccc} & A & C & G & T\\ A & * & f_{C}r_{AC} & f_{G}r_{AG} & f_{T}r_{AT}\\ C & f_{A}r_{AC} & * & f_{G}r_{CG} & f_{T}r_{CT}\\ G & f_{A}r_{AG} & f_{C}r_{CG} & * & f_{T}r_{GT}\\ T & f_{A}r_{AT} & f_{C}r_{CT} & f_{G}r_{GT} & * \end{array}\right]$$

GTR provides a high degree of flexibility and biological complexity by allowing all rates and frequencies to vary [25]. In some cases, it may be more suitable to use the HKY85 model (e.g., to prevent over-parameterization of limited data). This model distinguishes between transitions and transversions via the transition/transversion rate ratio (κ) [26]. In the simplest case, the Jukes Cantor (JC69) nucleotide substitution model assumes equal base frequencies and mutation rates [27]. Variation in the substitution rate across the genome can be modeled with a gamma distribution, which is often split into four discrete categories for computational efficiency [28].

## Step 2: Estimation of the Evolutionary Rate

The substitution or evolutionary rate parameter describes the frequency with which new mutations replace existing variants within a population (they become “fixed”). This parameter differs from the mutation rate, which describes the frequency with which mutations arise during DNA replication. The evolutionary rate can provide some indication of the adaptive potential of the population in response to environmental changes. It is often termed the “clock rate” in reference to the molecular clock hypothesis that substitutions arise regularly over time in a population [29]. The evolutionary rate is often assumed constant across all branches in the phylogenetic tree (a strict molecular clock), in which case the branch lengths are interpreted as proportional to the time that elapsed between the ancestor and descendant of each branch. Support for a strict clock can also be tested using the relative rates test, which compares the distance of each individual in a pair of taxa with a more distantly related taxon [30,31]. Otherwise, the evolutionary rate might be estimated per branch (a relaxed molecular clock [32]) to investigate differences in evolutionary rate across time or space [33,34].

If the sampling times of genome sequences are known, then the evolutionary rate can be calibrated in terms of substitutions per site per unit time. The evolutionary rate can be quickly estimated by plotting the sampling time of each isolate against the total branch distance to the root of the phylogenetic tree, provided the position of the root is accurate (Fig 1c and 1d). The date-randomization test repeatedly shuffles the sampling times across the tips to generate the rate distribution expected in the absence of any temporal signal. If the rate estimated with the correct sample times lies sufficiently outside this distribution, this is deemed as support for clock-like behavior. Bayesian phylogenetics approaches such as BEAST can model the evolutionary rate parameter on each branch of the tree, allowing estimation of the variation in evolutionary rate across branches and the uncertainty in parameter estimates [23,24]. Estimates of the evolutionary rate are often made under the assumption of neutral evolution. The presence of selection can distort branch lengths in the phylogenetic tree and lead to inaccurate estimates of the evolutionary rate.

## Step 3: Genome Annotation

Popular approaches to detecting selection rely on classification of substitutions according to their likely functional effect. This is discussed in Step 4, but first requires an interpretation of the genomic context in which substitutions occur, and this falls under the auspices of genome annotation. At its simplest, genome annotation involves prediction of coding sequences by identifying open reading frames (ORFs), which are regions of DNA sequence that encode a single polypeptide. However, sophisticated annotation pipelines now exist that perform a variety of functions that combine direct interpretation of the sequence with the borrowing or "lifting over" of annotations from other, better-studied reference genomes via searches for sequence similarity (homology).

Annotation can be carried out using a variety of Web-based or locally installed systems (reviewed by [35]), such as XBASE [36], GeneMark [37], GLIMMER [38,39], BASys [40], RAST [41], and Prokka [42]. The accuracy of automated genome annotation is dependent on several factors, including the accuracy of reference genome databases and the pseudogene content and quality of the query genome, meaning that manual checking is often necessary [35].

## Step 4: Classification of Substitutions

In order to perform basic tests for selection, it is necessary to classify all substitutions. At the most basic level, this can involve distinguishing protein-altering (non-synonymous) from non—protein-altering (synonymous) substitutions in coding regions. More sophisticated classification may further distinguish protein-truncating (nonsense) and intergenic (outside a coding region) substitutions, and it may sub-classify substitutions in coding regions by the function of the gene or non-coding substitutions by the regulatory function of the region or the distance from a gene [43,44].

When classifying substitutions, it helps to reconstruct ancestral sequences at internal nodes of the tree, which is usually carried out using parsimony or a probabilistic model of sequence evolution that returns the most likely ancestral sequences [45–47]. The programs FastML and PAML use maximum likelihood to perform ancestral sequence reconstruction for nucleotide, codon, or amino acid sequences [47–50]. The simplest method of classifying amino acid substitutions is to assume no more than a single nucleotide in the triplet changes along a branch. However, a more sophisticated approach is required when multiple sites in a codon may have undergone substitution. For these reasons, ML methods have been developed for estimating the number of synonymous and non-synonymous substitutions along a branch, which also account for variation in transition rates and base frequency [51,52].

## Step 5: Testing for Selection

Selection can act on genetic variation in different ways. In a simple model of directional selection, a novel mutation may be favored if it confers some sort of selective advantage to the bacterium (positive selection) or it may be disfavored if the mutation is deleterious to the bacterium (purifying or negative selection). Both positive and negative selection can be measured at individual amino acids, across genes or over the entire genome. Here, we outline three approaches that can be applied to divergent microbial populations in the absence of recombination to detect selection acting on genes in the population since their most recent common ancestor. When applying these methods to clonally evolving bacteria, it’s also important to consider how the tight linkage across sites can affect estimates of selection (for reviews, see [53,54]).

### A) Elevated substitution rates signal positive selection

**Fig 2:**
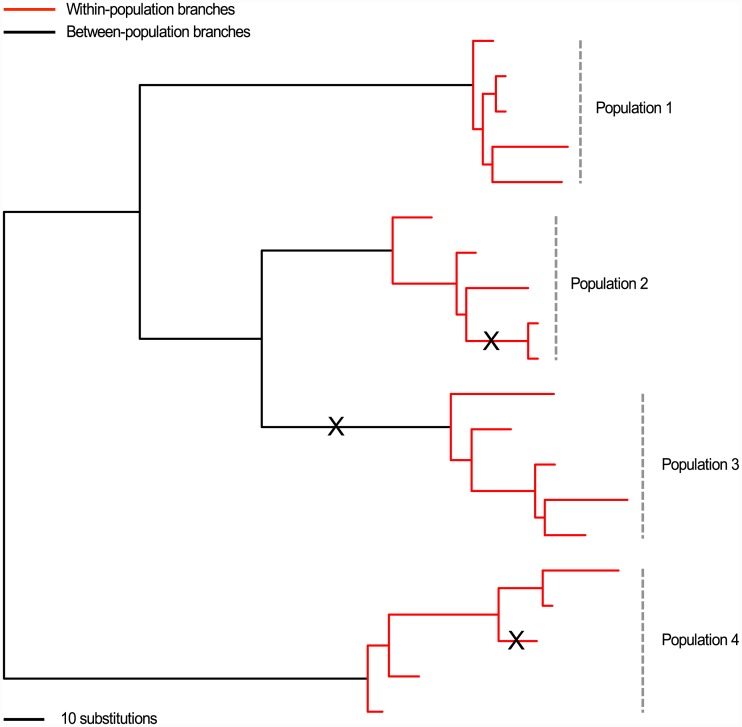
Detecting selection from microbial sequence data. The phylogeny shows the evolutionary history of 20 sequences sampled evenly from four divergent populations. d_N_/d_S_ methods test for selection by comparing the rates of non-synonymous and synonymous substitution occurring between divergent lineages (i.e., only substitutions that have occurred on the black branches) with those expected under neutrality. In contrast, the McDonald-Kreitman test for selection compares the ratio of non-synonymous and synonymous polymorphisms that are present within populations (due to substitutions occurring on red branches) with the ratio of non-synonymous and synonymous fixed differences that are present between populations (due to substitutions occurring on black branches). The phylogeny can also be used to detect selection by identifying parallel evolution, whereby recurrent mutations occur at a site or across a gene during the evolutionary history of a sample (for example, substitution X on the phylogeny).

Sites or genes are expected to mutate independently in microbial genomes within different individuals (or populations). Observing the recurrent emergence of the same substitution within different individuals is a signature of parallel or convergent evolution, most likely in response to a common selection pressure (Fig 2) [55]. For example, the selective pressure exerted on *Mycobacterium tuberculosis* by antimicrobial drugs during tuberculosis (TB) treatment is clearly identified by the frequent emergence of the same drug resistance point mutation within different patients [8]. Signals of positive selection may also manifest as numerous different substitutions across sites within a gene, given they are likely to have similar effects on the encoded protein [56]. The *rpoB* gene in *Mycobacterium tuberculosis* can mutate at several different sites within a “hot spot” region to confer resistance to the first-line anti-tuberculosis drug rifampicin [57].

Under the null hypothesis of neutral evolution, constant mutation rates across genes, and no recombination (*H*_*0*_), the number of substitutions per gene is expected to follow a Poisson process. The number of substitutions expected per gene can be calculated by multiplying the per-site mutation rate and the length of the gene. Any significant increase in the substitution rate of a gene from that expected under *H*_*0*_ can be used as support for positive selection having acted on the gene. However, an elevated substitution rate within a gene of interest may be due to a number of other factors, including variation in the mutation rate across genes or recombination. Therefore, more commonly used methods for detecting positive selection look for a significant difference in the rate of substitutions that have a functional effect on the protein relative to those that do not.

### B) Estimates of d_N_/d_S_

Comparison of the rate of non-synonymous substitution per non-synonymous site (d_N_) to the rate of synonymous substitution per synonymous site (d_S_) is a popular method of detecting selection between divergent populations [58,59]. Due to the redundancy of the genetic code, random mutations generate a greater number of non-synonymous than synonymous substitutions. In order to estimate d_N_/d_S_, the ratio of raw counts of non-synonymous and synonymous substitutions must be adjusted by the ratio that one would expect to see in the absence of any selection (i.e., under strict neutrality). The null hypothesis (*H*_*0*_) is that the ratio of non-synonymous and synonymous counts does not significantly differ from the ratio expected by chance (*r*_*0*_). This means that when d_N_/d_S_ is close to one, it is inferred to be evolving strictly neutrally, in the absence of selection. Estimates >1 suggest that positive selection has acted on the sequence, while those <1 are indicative of negative selection.

The estimate of d_N_/d_S_ under the null hypothesis can be obtained via calculation of the codon substitution rate matrix, which describes the rate of substitution from one codon to another. The Nielsen and Yang (NY98) model of codon substitution is similar to the HKY85 model of nucleotide substitution, in that it allows both codon frequencies and the rates of transitions and transversions to vary [59]. Since there are many more codons than bases, the NY98 model is described by a (61 × 61) *Q* matrix (rather than the 4 × 4 HKY85 matrix above), which includes the probability of transitions between all pairs of amino-acid codons (rather than nucleotides). The model includes a parameter ω, representing the value of d_N_/d_S_ and κ, the transition/transversion rate ratio. Rather than drawing the entire rate matrix for the NY98 model, we can describe it for a given pair of codons *i* and *j*, as:

$$Q_{i}{}_{j}=\{\begin{array}{ll} 0, & \text{if the two codons differ at more than one position}\\ f_{j}, & \text{for synonymous transversion}\\ \kappa f_{j}, & \text{for synonymous transition}\\ \omega f_{j}, & \text{for non-synonymous transversion}\\ \omega \kappa f_{j}, & \text{for non-synonymous transition} \end{array}$$

The codon frequencies, *f*, are often estimated directly from the sequence data, while κ can be estimated using maximum likelihood approaches, such as those implemented in the phylogenetics software PhyML [21]. Either ω can be estimated formally and tested against the null hypothesis that it equals one under neutrality, or the expected ratio *r*_*0*_ of non-synonymous and synonymous counts can be computed under neutrality and compared to the observed counts from Step 4 to test for any signal of positive or negative selection.

However, application of d_N_/d_S_ methods to microbial populations is complicated by several factors. Firstly, the test may be statistically underpowered for detecting non-neutral d_N_/d_S_ per site if the number of substitutions expected at any individual position is small. Usually it is more powerful to sum substitutions across sites in the same gene to estimate a per-gene d_N_/d_S_, which can reveal whether selection has acted differently across genes. Secondly, the existence of sites subject to negative selection is highly likely in any functional protein-coding sequence, and these sites reduce the true value of d_N_/d_S_ to below the value of one predicted under the strict neutrality hypothesis. The presence of sites subject to negative selection reduces the probability, and hence statistical power, to detect positive selection even when it is present.

Thirdly, the d_N_/d_S_ statistic assumes that differences between lineages are fixed (i.e., that lineages have been diverging for a long time), while substitutions between isolates sampled from closely related microbial populations (e.g., between hosts in an outbreak) are likely to represent segregating polymorphisms [60]. Within-population microbial variation has often arisen relatively recently and due to the evolutionary time-lag, selection may not yet have had time to purge deleterious mutations and fix beneficial mutations. Therefore patterns of polymorphism are expected to appear more neutral (d_N_/d_S_ closer to one) than patterns of fixation. Over time, slightly deleterious non-synonymous substitutions are purged from the population, so estimates of d_N_/d_S_ tend to decrease as sampled microbial lineages diverge from their most recent common ancestor [60]. The McDonald-Kreitman test, described in the next section, takes advantage of this phenomenon by comparing the divergence between lineages with the polymorphism within them, giving it greater power to detect selection [61].

### C) The McDonald-Kreitman test

The McDonald-Kreitman (MK) test tests for non-neutral evolution by comparing the ratio of non-synonymous to synonymous polymorphisms within a species (*P*_n_/*P*_s_) to the ratio of non-synonymous to synonymous fixed differences between species (*D*_n_/*D*_s_) (Fig 2) [61]. It compares the ratios of raw counts without directly calculating a d_N_/d_S_ ratio. Although it is often applied to test for selection within species, it can also be applied to sub-populations (e.g., comparing within and between host rates of substitution). The test is set up with a two-way contingency table (Table 1).

**Table 1:** Two-way contingency table used in the MacDonald-Kreitman test.

	Fixed differences	Polymorphisms
Synonymous mutations	*D*_s_	*P*_s_
Non-synonymous mutations	*D*_n_	*P*_n_

*D*_n_/*D*_s_ > *P*_n_/*P*_s_, indicates an excess of non-synonymous changes among the fixed differences distinguishing the two groups, thus implying positive selection. *D*_n_/*D*_s_ < *P*_n_/*P*_s_ represents a paucity of non-synonymous fixed differences between groups, indicating their removal by purifying selection. The proportion of non-synonymous substitutions (α) under positive selection can be calculated for each gene individually, or a genome-wide estimate of α can be obtained by averaging these count data across genes [62].

The MK test is robust to variation in the mutation rate and evolutionary histories across sites in the genome [63]. However, the presence of mildly deleterious mutations that are not immediately purged from the population increases *P*_n_/*P*_s_ and reduces estimates of α, leading to loss of power to detect positive selection. Extensions of the MK test attempt to remove the effect of mildly deleterious mutations by excluding polymorphisms segregating at low frequencies from the analysis [64,65].

## Conclusions

This tutorial has demonstrated how basic population genetics methods can be applied to microbial WGS data to learn about their evolutionary history and the selective pressures acting on them. The methods presented here and in the accompanying exercise (S1 File) have not attempted to address analysis of selection in recombining bacteria. In analyses that rely on estimation of phylogenetic trees, homologous recombination and horizontal gene transfer risk causing false detection of positive selection [66–68]. Several methods are available for detecting such processes (for reviews see [69–71]), while new methods developed specifically for application to whole bacterial genomes are also now available [72–74].

## Supporting Information

S1 File Exercise: Practical approaches for detecting within-host selection in *Burkholderia dolosa*. Compressed file containing all material for the exercise, including the description of the exercises and input data files.
(ZIP)

S1 Table Microbial genomics applied. A selection of published analyses employing the methods described in Steps 1–5 to address a range of evolutionary questions across different microbial species.
(PDF)

## References

[1] Didelot X, Bowden R, Wilson DJ, Peto TEA, Crook DW. Transforming clinical microbiology with bacterial genome sequencing. Nat Rev Genet. 2012;13(9):601–12. 10.1038/nrg3226 22868263 PMC5049685

[2] Stratton MR. Exploring the genomes of cancer cells: progress and promise. Science. 2011;331(6024):1553–8. 10.1126/science.1204040 21436442

[3] Green ED, Guyer MS. Charting a course for genomic medicine from base pairs to bedside. Nature. 2011;470(7333):204–13. 10.1038/nature09764 21307933

[4] Lieberman TD, Michel J-B, Aingaran M, Potter-Bynoe G, Roux D, Davis MR, et al Parallel bacterial evolution within multiple patients identifies candidate pathogenicity genes. Nat Genet. 2011;43(12):1275–80. 10.1038/ng.997 22081229 PMC3245322

[5] Pepperell CS, Casto AM, Kitchen A, Granka JM, Cornejo OE, Holmes EC, et al The role of selection in shaping diversity of natural M. tuberculosis populations. PLoS Pathog. 2013;9(8):e1003543 10.1371/journal.ppat.1003543 23966858 PMC3744410

[6] McAdam PR, Templeton KE, Edwards GF, Holden MTG, Feil EJ, Aanensen DM, et al Molecular tracing of the emergence, adaptation, and transmission of hospital-associated methicillin-resistant Staphylococcus aureus. Proc Natl Acad Sci. 2012;109(23):9107–12. 10.1073/pnas.1202869109 22586109 PMC3384211

[7] Holt KE, Parkhill J, Mazzoni CJ, Roumagnac P, Weill F-X, Goodhead I, et al High-throughput sequencing provides insights into genome variation and evolution in Salmonella Typhi. Nat Genet. 2008;40(8):987–93. 10.1038/ng.195 18660809 PMC2652037

[8] Farhat MR, Shapiro BJ, Kieser KJ, Sultana R, Jacobson KR, Victor TC, et al Genomic analysis identifies targets of convergent positive selection in drug-resistant Mycobacterium tuberculosis. Nat Genet. 2013;45(10):1183–9. 10.1038/ng.2747 23995135 PMC3887553

[9] Holden MTG, Hsu L, Kurt K, Weinert L a, Mather AE, Harris SR, et al A genomic portrait of the emergence, evolution and global spread of a methicillin resistant Staphylococcus aureus pandemic. Genome Res. 2013;23(4):653–64. 10.1101/gr.147710.112 23299977 PMC3613582

[10] Azarian T, Ali A, Johnson JA, Mohr D, Prosperi M, Veras NM, et al Phylodynamic Analysis of Clinical and Environmental Vibrio cholerae Isolates from Haiti Reveals Diversification Driven by Positive Selection. MBio. 2014;5(6):e01824–14. 10.1128/mBio.01824-14 25538191 PMC4278535

[11] Comas I, Chakravartti J, Small PM, Galagan J, Niemann S, Kremer K, et al Human T cell epitopes of Mycobacterium tuberculosis are evolutionarily hyperconserved. Nat Genet. 2010;42(6):498–503. 10.1038/ng.590 20495566 PMC2883744

[12] Mena A, Smith EE, Burns JL, Speert DP, Moskowitz SM, Perez JL, et al Genetic adaptation of Pseudomonas aeruginosa to the airways of cystic fibrosis patients is catalyzed by hypermutation. J Bacteriol. 2008;190(24):7910–7. 10.1128/JB.01147-08 18849421 PMC2593214

[13] Edwards DJ, Holt KE. Beginner’s guide to comparative bacterial genome analysis using next-generation sequence data. Microb Inform Exp. 2013;3(1):2 10.1186/2042-5783-3-2 23575213 PMC3630013

[14] Loman NJ, Constantinidou C, Chan JZM, Halachev M, Sergeant M, Penn CW, et al High-throughput bacterial genome sequencing: an embarrassment of choice, a world of opportunity. Nat Rev Microbiol. 2012;10(9):599–606. 10.1038/nrmicro2850 22864262

[15] Harris SR, Feil EJ, Holden MTG, Quail MA, Nickerson EK, Chantratita N, et al Evolution of MRSA during hospital transmission and intercontinental spread. Science. 2010;327(5964):469–74. 10.1126/science.1182395 20093474 PMC2821690

[16] Gardy JL, Johnston JC, Ho Sui SJ, Cook VJ, Shah L, Brodkin E, et al Whole-genome sequencing and social-network analysis of a tuberculosis outbreak. N Engl J Med. 2011;364(8):730–9. 10.1056/NEJMoa1003176 21345102

[17] Chin C-S, Sorenson J, Harris JB, Robins WP, Charles RC, Jean-Charles RR, et al The origin of the Haitian cholera outbreak strain. N Engl J Med. 2011;364(1):33–42. 10.1056/NEJMoa1012928 21142692 PMC3030187

[18] Felsenstein J. Inferring Phylogenies. 1st ed Sunderland, Massachusetts, USA: Sinauer Associates, Inc.; 2004.

[19] Lemey P, Salemi M, Vandamme A-M, editors. The Phylogenetic Handbook. 2nd ed Cambridge, UK: Cambridge University Press; 2009.

[20] Baldauf SL. Phylogeny for the faint of heart: A tutorial. Trends Genet. 2003;19(6):345–51. 12801728 10.1016/S0168-9525(03)00112-4

[21] Guindon S, Gascuel O. A simple, fast, and accurate algorithm to estimate large phylogenies by maximum likelihood. Syst Biol. 2003;52(5):696–704. 14530136 10.1080/10635150390235520

[22] Stamatakis A. RAxML version 8: A tool for phylogenetic analysis and post-analysis of large phylogenies. Bioinformatics. 2014;30(9):1312–3. 10.1093/bioinformatics/btu033 24451623 PMC3998144

[23] Drummond AJ, Suchard MA, Xie D, Rambaut A. Bayesian phylogenetics with BEAUti and the BEAST 1.7. Mol Biol Evol. 2012;29(8):1969–73. 10.1093/molbev/mss075 22367748 PMC3408070

[24] Drummond AJ, Rambaut A. BEAST: Bayesian evolutionary analysis by sampling trees. BMC Evol Biol. 2007;7:214 17996036 10.1186/1471-2148-7-214 PMC2247476

[25] Tavare S. Some probabilistic and statistical problems in the analysis of DNA sequences In: American Mathematical Society: Lectures on Mathematics in the Life Sciences. 1986 p. 57–86.

[26] Hasegawa M, Kishino H, Yano T aki. Dating of the human-ape splitting by a molecular clock of mitochondrial DNA. J Mol Evol. 1985;22(2):160–74. 3934395 10.1007/BF02101694

[27] Jukes TH, Cantor CR. Evolution of protein molecules In: Munro HN, editor. Mammalian Protein Metabolism. New York: Academic Press; 1969 p. 21–132.

[28] Yang Z. Maximum likelihood phylogenetic estimation from DNA sequences with variable rates over sites: approximate methods. J Mol Evol. 1994;39(3):306–14. 7932792 10.1007/BF00160154

[29] Zuckerkandl E, Pauling L. Molecular disease, evolution and genetic heterogeneity In: Kasha M, Pullman B, editors. Horizons in Biochemistry. 1st ed New York: Academic Press; 1962 p. 189–225.

[30] Wu CI, Li WH. Evidence for higher rates of nucleotide substitution in rodents than in man. Proc Natl Acad Sci. 1985;82(6):1741–5. 3856856 10.1073/pnas.82.6.1741 PMC397348

[31] Sarich VM, Wilson AC. Generation time and genomic evolution in primates. Science. 1973;179(4078):1144–7. 4120260 10.1126/science.179.4078.1144

[32] Drummond AJ, Ho SYW, Phillips MJ, Rambaut A. Relaxed phylogenetics and dating with confidence. PLoS Biol. 2006;4(5):699–710. 10.1371/journal.pbio.0040088 PMC1395354 16683862

[33] Cui Y, Yu C, Yan Y, Li D, Li Y, Jombart T, et al Historical variations in mutation rate in an epidemic pathogen, Yersinia pestis. Proc Natl Acad Sci. 2013;110(2):577–82. 10.1073/pnas.1205750110 23271803 PMC3545753

[34] Morelli G, Didelot X, Kusecek B, Schwarz S, Bahlawane C, Falush D, et al Microevolution of Helicobacter pylori during prolonged infection of single hosts and within families. PLoS Genet. 2010;6(7):e1001036 10.1371/journal.pgen.1001036 20661309 PMC2908706

[35] Richardson EJ, Watson M. The automatic annotation of bacterial genomes. Brief Bioinform. 2013;14(1):1–12. 10.1093/bib/bbs007 22408191 PMC3548604

[36] Chaudhuri RR, Pallen MJ. xBASE, a collection of online databases for bacterial comparative genomics. Nucleic Acids Res. 2006;34:D335–7. 16381881 10.1093/nar/gkj140 PMC1347502

[37] Besemer J, Borodovsky M. GeneMark: Web software for gene finding in prokaryotes, eukaryotes and viruses. Nucleic Acids Res. 2005;33(SUPPL. 2):W451–4. 15980510 10.1093/nar/gki487 PMC1160247

[38] Delcher AL, Harmon D, Kasif S, White O, Salzberg SL. Improved microbial gene identification with GLIMMER. Nucleic Acids Res. 1999;27(23):4636–41. 10556321 10.1093/nar/27.23.4636 PMC148753

[39] Delcher AL, Bratke KA, Powers EC, Salzberg SL. Identifying bacterial genes and endosymbiont DNA with Glimmer. Bioinformatics. 2007;23(6):673–9. 17237039 10.1093/bioinformatics/btm009 PMC2387122

[40] Van Domselaar GH, Stothard P, Shrivastava S, Cruz JA, Guo AC, Dong X, et al BASys: A web server for automated bacterial genome annotation. Nucleic Acids Res. 2005;33(SUPPL. 2):W455–9. 15980511 10.1093/nar/gki593 PMC1160269

[41] Aziz RK, Bartels D, Best AA, DeJongh M, Disz T, Edwards RA, et al The RAST Server: rapid annotations using subsystems technology. BMC Genomics. 2008;9(1):75. 18261238 10.1186/1471-2164-9-75 PMC2265698

[42] Seemann T. Prokka: Rapid prokaryotic genome annotation. Bioinformatics. 2014;30(14):2068–9. 10.1093/bioinformatics/btu153 24642063

[43] Young BC, Golubchik T, Batty EM, Fung R, Larner-Svensson H, Votintseva AA, et al Evolutionary dynamics of Staphylococcus aureus during progression from carriage to disease. Proc Natl Acad Sci. 2012;109(12):4550–5. 10.1073/pnas.1113219109 22393007 PMC3311376

[44] Zhang H, Li D, Zhao L, Fleming J, Lin N, Wang T, et al Genome sequencing of 161 Mycobacterium tuberculosis isolates from China identifies genes and intergenic regions associated with drug resistance. Nat Genet. 2013;45(10):1255–60. 10.1038/ng.2735 23995137

[45] Yang Z, Kumar S, Nei M. A new method of inference of ancestral nucleotide and amino acid sequences. Genetics. 1995;141:1641–50. 8601501 10.1093/genetics/141.4.1641 PMC1206894

[46] Koshi JM, Goldstein RA. Probabilistic reconstruction of ancestral protein sequences. J Mol Evol. 1996;42(2):313–20. 8919883 10.1007/BF02198858

[47] Pupko T, Pe’er I, Shamir R, Graur D. A fast algorithm for joint reconstruction of ancestral amino acid sequences. Mol Biol Evol. 2000;17(6):890–6. 10833195 10.1093/oxfordjournals.molbev.a026369

[48] Yang Z. PAML 4: Phylogenetic Analysis by Maximum Likelihood. Mol Biol Evol. 2007;24(8):1586–91. 17483113 10.1093/molbev/msm088

[49] Yang Z. PAML: a program package for phylogenetic analysis by maximum likelihood. Comput Appl Biosci. 1997;13(5):555–6. 9367129 10.1093/bioinformatics/13.5.555

[50] Ashkenazy H, Penn O, Doron-Faigenboim A, Cohen O, Cannarozzi G, Zomer O, et al FastML: A web server for probabilistic reconstruction of ancestral sequences. Nucleic Acids Res. 2012;40:W580–4. 10.1093/nar/gks498 22661579 PMC3394241

[51] Yang Z, Nielsen R. Estimating synonymous and nonsynonymous substitution rates under realistic evolutionary models. Mol Biol Evol. 2000;17(1):32–43. 10666704 10.1093/oxfordjournals.molbev.a026236

[52] Goldman N, Yang Z. A codon-based model of nucleotide substitution for protein-coding DNA sequences. Mol Biol Evol. 1994;11(5):725–36. 7968486 10.1093/oxfordjournals.molbev.a040153

[53] Charlesworth B. The effects of deleterious mutations on evolution at linked sites. Genetics. 2012;190(1):5–22. 10.1534/genetics.111.134288 22219506 PMC3249359

[54] Sniegowski PD, Gerrish PJ. Beneficial mutations and the dynamics of adaptation in asexual populations. Philos Trans R Soc B. 2010;365(1544):1255–63. 10.1098/rstb.2009.0290 PMC2871819 20308101

[55] Chattopadhyay S, Weissman SJ, Minin VN, Russo TA, Dykhuizen DE, Sokurenko E V. High frequency of hotspot mutations in core genes of Escherichia coli due to short-term positive selection. Proc Natl Acad Sci. 2009;106(30):12412–7. 10.1073/pnas.0906217106 19617543 PMC2718352

[56] Woods R, Schneider D, Winkworth CL, Riley MA, Lenski RE. Tests of parallel molecular evolution in a long-term experiment with Escherichia coli. Proc Natl Acad Sci. 2006;103(24):9107–12. 16751270 10.1073/pnas.0602917103 PMC1482574

[57] Gagneux S, Long CD, Small PM, Van T, Schoolnik GK, Bohannan BJM. The competitive cost of antibiotic resistance in Mycobacterium tuberculosis. Science. 2006;312(5782):1944–6. 16809538 10.1126/science.1124410

[58] Smith NH, Maynard Smith J, Spratt BG. Sequence evolution of the porB gene of Neisseria gonorrhoeae and Neisseria meningitidis: evidence of positive Darwinian selection. Mol Biol Evol. 1995;12(3):363–70. 7739379 10.1093/oxfordjournals.molbev.a040212

[59] Nielsen R, Yang Z. Likelihood models for detecting positively selected amino acid sites and applications to the HIV-1 envelope gene. Genetics. 1998;148(3):929–36. 9539414 10.1093/genetics/148.3.929 PMC1460041

[60] Rocha EPC, Smith JM, Hurst LD, Holden MTG, Cooper JE, Smith NH, et al Comparisons of dN/dS are time dependent for closely related bacterial genomes. J Theor Biol. 2006;239(2):226–35. 16239014 10.1016/j.jtbi.2005.08.037

[61] McDonald JH, Kreitman M. Adaptive protein evolution at the Adh locus in Drosophila. Nature. 1991;351(6328):652–4. 1904993 10.1038/351652a0

[62] Smith NGC, Eyre-Walker A. Adaptive protein evolution in Drosophila. Nature. 2002;415:1022–4. 11875568 10.1038/4151022a

[63] Bierne N, Eyre-Walker A. The genomic rate of adaptive amino acid substitution in Drosophila. Mol Biol Evol. 2004;21(7):1350–60. 15044594 10.1093/molbev/msh134

[64] Charlesworth J, Eyre-Walker A. The McDonald-Kreitman test and slightly deleterious mutations. Mol Biol Evol. 2008;25(6):1007–15. 10.1093/molbev/msn005 18195052

[65] Fay JC, Wyckoff GJ, Wu CI. Positive and negative selection on the human genome. Genetics. 2001;158(3):1227–34. 11454770 10.1093/genetics/158.3.1227 PMC1461725

[66] Anisimova M, Nielsen R, Yang Z. Effect of recombination on the accuracy of the likelihood method for detecting positive selection at amino acid sites. Genetics. 2003;164(3):1229–36. 12871927 10.1093/genetics/164.3.1229 PMC1462615

[67] Shriner D, Nickle DC, Jensen MA, Mullins JI. Potential impact of recombination on sitewise approaches for detecting positive natural selection. Genet Res. 2003;81(2):115–21. 12872913 10.1017/s0016672303006128

[68] Arenas M, Posada D. The influence of recombination on the estimation of selection from coding sequence alignments In: Faras MA, editor. Natural Selection: Methods and Applications. CRC Press/Taylor & Francis; 2014 p. 112–21.

[69] Posada D, Crandall KA, Holmes EC. Recombination in evolutionary genomics. Annu Rev Genet. 2002;36:75–97. 12429687 10.1146/annurev.genet.36.040202.111115

[70] Salminen M, Martin D. Detecting and characterizing individual recombination events In: Lemey P, Salemi M, Vandamme A-M, editors. The Phylogenetic Handbook. 1st ed Cambridge, UK: Cambridge University Press; 2009 p. 519–48.

[71] Awadalla P. The evolutionary genomics of pathogen recombination. Nat Rev Genet. 2003;4(1):50–60. 12509753 10.1038/nrg964

[72] Didelot X, Wilson DJ. ClonalFrameML: Efficient Inference of Recombination in Whole Bacterial Genomes. PLoS Comput Biol. 2015;11(2):e1004041 10.1371/journal.pcbi.1004041 25675341 PMC4326465

[73] Croucher NJ, Page AJ, Connor TR, Delaney AJ, Keane JA, Bentley SD, et al Rapid phylogenetic analysis of large samples of recombinant bacterial whole genome sequences using Gubbins. Nucleic Acids Res. 2014;43(3):e15 10.1093/nar/gku1196 25414349 PMC4330336

[74] Marttinen P, Hanage WP, Croucher NJ, Connor TR, Harris SR, Bentley SD, et al Detection of recombination events in bacterial genomes from large population samples. Nucleic Acids Res. 2012;40(1):1–12. 22064866 10.1093/nar/gkr928 PMC3245952

