# Supplementary material for: Practical Approaches for Detecting Selection in Microbial Genomes
Source: PLoS Comput Biol. 2016 Feb 11;12(2):e1004739. doi: 10.1371/journal.pcbi.1004739 (PMC4750996; doi:10.1371/journal.pcbi.1004739)
Supplement: S1 File — Compressed file containing all material for the exercise, including the description of the exercises and input data files. (ZIP) [file pcbi.1004739.s001.zip › S1_file/microbialEvolutionExercise.pdf]

Exercise: Practical Approaches for Detecting Within-Host Selection in *Burkholderia dolosa* Genomes

This exercise will guide you through several common steps in the analysis of the evolution and selection acting on microbial whole genome sequences (WGS). This includes phylogenetic tree construction, molecular clock rate estimation, genome annotation and three approaches for detecting signals of selection. The majority of the analyses will be performed in the R programming language and the corresponding code (in grey boxes), comments (marked with #) and output (marked with ##) are provided at each step. However some commands should be run from the command line and this will be specified in the text (e.g. creating directories and running software).

We will be analysing WGS data of the bacterium *Burkholderia dolosa*, which opportunistically infects the airways of people with cystic fibrosis. The data was published by Lieberman *et al.* (2011) *Nature Genetics* 43(12) 1275-1280(1) and comprises 112 isolates that were sampled during a small epidemic in the 1990s in Boston. These isolates were collected over a period of 16 years from 14 patients, including the first identified case of the epidemic (the index case).

The following files are provided as part of this exercise in the folder "inputData":

- Lieberman_etal_variants.tab.txt: the variant sites file (provided as Supplementary Table 2 in (1))
- B.dolosa.AU0158.fa: *B. dolosa* reference genome sequence in FASTA format
- Codon_change_types.txt: a matrix defining types of substitutions

Ensure that you have the following software required for this exercise installed on your computer:

- R: http://www.r-project.org
- PhyML: http://atgc.lirmm.fr/phyml/binaries.php
- FigTree: http://tree.bio.ed.ac.uk/software/figtree
- Artemis: http://www.sanger.ac.uk/science/tools/artemis
- FastML: http://fastml.tau.ac.il/source.php

For the example provided here, the sequence data must be read in and some initial pre-processing carried out prior to the phylogenetic analysis performed in Step 1. Step 0 is therefore unnecessary in analyses for which a multiple sequence alignment is already available in FASTA and PHYLIP formats.

STEP 0: Data formatting

First, set the working directory to the location of the two data files provided in the folder called "Supporting_Information_S1":

```
# Set the working directory to the folder Supporting_Information_S1, for example:
setwd("~/Documents/S1_file")
```

Bioinformatics pipelines typically output only the details of sites that were variable in the sample to save space. However, to correctly estimate substitution rates, the invariant sites must be included in the data prior to estimating the phylogenetic tree. Failing to include the invariant sites is a common mistake that can mislead phylogenetic reconstruction. We will start by reading in the variant sites file:

```r
# Read the text file
variants = read.delim("inputData/Lieberman_etal_variants.tab.txt",as.is=TRUE)
# R sometimes replaces some column names with certain characters, so read in separately
varHeader = scan("inputData/Lieberman_etal_variants.tab.txt",
               what=character(0),nlines=1,sep="\t")
# Preview the data
head(variants)
```

```
##   Chromosome..Position Category..CDS.coding.sequence..INT.intergenic.
## 1              1::5666                                            CDS
## 2              1::8477                                            CDS
## 3             1::22647                                            INT
## 4             1::23774                                            INT
## 5             1::26068                                            INT
## 6             1::51329                                            CDS
##   Gene..when.intergenic..nucleotides.upstream..if.smaller.than.75bp.
## 1                                                         BDAG_00006
## 2                                                         BDAG_00009
## 3
## 4
## 5
## 6                                                         BDAG_00045
##   Annotation..Broad.Institute..ref.30.of.main.text.
## 1                              Flavodoxin reductase
## 2                   Histone acetyltransferase HPA6
## 3
## 4
## 5
## 6                      H-NS histone family protein
##   Number.of.mutations.across.the.phylogeny AU0158..reference.
## 1                                        1                  C
## 2                                        1                  G
## 3                                        1                  C
## 4                                        1                  A
## 5                                        1                  A
## 6                                        1                  G
##   Last.common.ancestor X.3.7 A.0.0 A.4.2a A.4.2b A.5.0a A.5.0b B.4.11
## 1                    C     C     C      C      C      C      C      C
## 2                    G     G     G      G      G      G      G      G
## 3                    C     C     C      C      C      C      C      C
## 4                    A     A     A      A      A      A      A      A
## 5                    A     A     A      A      A      A      A      A
## 6                    G     G     T      G      G      G      G      G
##   B.7.1 B.9.1 B.11.1 C.4.11 C.8.0 C.10.0 C.12.1 D.7.10 E.6.1 E.8.4 E.9.5
## 1     C     C      C      C     C      C      C      C     C     C     C
## 2     G     G      G      G     G      G      G      G     G     G     G
## 3     C     C      C      C     C      C      C      C     C     C     C
## 4     A     A      A      A     A      A      A      A     A     A     A
## 5     A     A      A      A     A      A      A      A     A     A     A
## 6     G     G      G      G     G      G      G      G     G     G     G
##   E.12.1 E.13.0 F.6.9 F.6.11 F.7.0a.. G.7.5 G.9.8 G.9.11b G.10.3 G.10.6
## 1      C      C     C      C        C     C     C       C      C      C
## 2      G      G     G      G        G     G     G       G      G      G
```

```
## 3      C      C     C      C        C     C     C       C      C      C
## 4      A      A     A      A        A     A     A       A      A      A
## 5      A      A     A      A        A     A     A       A      A      A
## 6      G      G     G      G        G     G     G       G      G      G
##   G.9.11a G.9.11c G.10.0a G.10.0b G.10.0c G.10.1b G.10.2 G.10.1c..
## 1       C       C       C       C       C       C      C         C
## 2       G       G       G       G       G       G      G         G
## 3       C       C       C       C       C       C      C         C
## 4       A       A       A       A       A       A      A         A
## 5       A       A       A       A       A       A      A         A
## 6       G       G       G       G       G       G      G         G
##   G.10.11b H.8.3 H.9.5 H.10.1 H.10.6a H.10.6b H.9.10 H.9.11 H.10.10 H.11.3
## 1        C     C     C      C       C       C      C      C       C      C
## 2        G     G     G      G       G       G      G      G       G      G
## 3        C     C     C      C       C       C      C      C       C      C
## 4        A     A     A      A       A       A      A      A       A      A
## 5        A     A     A      A       A       A      A      A       A      A
## 6        G     G     G      G       G       G      G      G       G      G
##   H.12.7 H.12.10 H.12.11a.. H.13.0b.. I.8.7 I.9.6 J.9.11 J.11.8 J.11.11
## 1      C       C          C         C     C     C      C      C       C
## 2      G       G          G         G     G     G      G      G       G
## 3      C       C          C         C     C     C      C      C       C
## 4      A       A          A         A     A     A      A      A       A
## 5      A       A          A         A     A     A      A      A       A
## 6      G       G          G         G     G     G      G      G       G
##   J.12.4 J.12.7 J.12.11 J.13.6a.. J.13.6b J.14.2 J.14.8 K.9.0 K.10.0
## 1      C      C       C         C       C      C      C     C      C
## 2      G      G       G         G       G      G      G     G      G
## 3      C      C       C         C       C      C      C     C      C
## 4      A      A       A         A       A      A      A     A      A
## 5      A      A       A         A       A      A      A     A      A
## 6      G      G       G         G       G      G      G     G      G
##   K.10.1 K.10.7 K.10.2 K.10.3a K.10.10 K.11.6 K.11.3a K.11.3b K.12.5
## 1      C      C      C       C       C      C       C       C      C
## 2      G      G      G       G       G      G       G       T      G
## 3      C      C      C       C       C      C       C       C      C
## 4      A      A      A       A       A      A       A       A      A
## 5      A      A      A       A       A      A       A       A      A
## 6      G      G      G       G       G      G       G       G      G
##   K.12.8a.. K.12.0a K.12.0b K.12.9.. L.9.11 L.10.1 L.10.2 L.10.6 L.10.10
## 1         C       C       C        C      C      C      C      C       C
## 2         G       G       G        G      G      G      G      G       G
## 3         C       G       C        C      C      C      C      C       C
## 4         A       C       A        A      A      A      A      A       A
## 5         A       A       A        A      A      A      A      A       A
## 6         G       G       G        G      G      G      G      G       G
##   L.11.3 L.11.6 L.11.8 L.11.11 L.12.3 L.12.7 L.13.0 L.13.7 L.13.4a..
## 1      C      C      C       C      C      C      C      C         G
## 2      G      G      G       G      G      G      G      G         G
## 3      C      C      C       C      C      C      C      C         C
## 4      A      A      A       A      A      A      A      A         A
## 5      A      A      A       A      A      A      A      A         A
## 6      G      G      G       G      G      G      G      G         G
##   L.16.0 M.9.4 M.10.6 M.11.5 M.12.6 N.10.0 N.10.1 N.10.5 N.10.8 N.10.11
```

```
## 1      C     C      C      C      C      C      C      C      C       C
## 2      G     G      G      G      G      G      G      G      G       G
## 3      C     C      C      C      C      C      C      C      C       C
## 4      A     A      A      A      A      A      A      A      A       A
## 5      A     G      G      G      G      A      A      A      A       A
## 6      G     G      G      G      G      G      G      G      G       G
##   N.11.2 N.11.3 N.11.7 N.11.9 N.12.1 N.12.6a N.12.6c N.12.6d.. N.12.6b
## 1      C      C      C      C      C       C       C         C       C
## 2      G      G      G      G      G       G       G         G       G
## 3      C      C      C      C      C       C       C         C       C
## 4      A      A      A      A      A       A       A         A       A
## 5      A      A      A      A      A       A       A         A       A
## 6      G      G      G      G      G       G       G         G       G
##   N.12.4a N.12.5.. N.12.4c..
## 1       C        C         C
## 2       G        G         G
## 3       C        C         C
## 4       A        A         A
## 5       A        A         A
## 6       G        G         G
```

The variants were discovered by mapping 70 bp single-ended Illumina reads to the closely related *Burkholderia dolosa* AU0158 reference genome. Write two functions that read in the reference genome in FASTA format as: (i) a single genome and (ii) the genome sequence split across chromosomes or contigs.

```r
# Assumes a fasta file representing a single genome
readFastaRef = function(refFile) {
  row = scan(refFile,what=character(0),sep="\n")
  # Convert each base to an individual character
    chars = substr(row,1,1)
    base = chars!=">"
    seq = paste(row[base],collapse="")
    return(toupper(unlist(strsplit(seq,""))))
}
# Assumes a fasta file representing a single genome, split across chromosomes/contigs
readFastaRefContig = function(refFile) {
    row = scan(refFile,what=character(0),sep="\n")
    chars = substr(row,1,1)
    base = chars!=">"
    chromNumber = rep(cumsum(!base)[base],times=nchar(row[base]))
    return(chromNumber)
}
# Read the reference genome
AU0158 = readFastaRef("inputData/B.dolosa.AU0158.fa")
# Get chromosome number per site
AU0158c = readFastaRefContig("inputData/B.dolosa.AU0158.fa")
table(AU0158) # Number of sites of A, C, G, T
```

```
## AU0158
##       A       C       G       N       T
## 1036598 2087596 2086689  172806 1036711
```

```
chromLen = table(AU0158c) # Number of sites in chromosomes 1, 2 and 3
```

Another common error made by variant detection pipelines that process genomes individually, rather than together as a sample of genomes, is to output information only about sites that can be confidently called different to the reference, and fail to output information about sites that cannot be confidently called. In downstream analysis, if the positions of ambiguous sites are unknown, they might well be assumed –mistakenly– to equal the reference, seriously misleading inference.

In the current dataset, the patient IDs and sampling times are encoded in the sequence names: a sequence labeled A-4-2b means that it was the second (b) sample from patient A in year 4, month 2. Some labels are suffixed with a $ to indicate bloodstream isolates. In order to simplify the sequence name, we will change the sequence name format to patient+sample_year, i.e. Ab_4.167 (this will help when estimating the evolutionary rate in Step 3 where sampling dates are obtained from the sequence names in the Path-O-Gen software).

Replace all ambiguous sites with an "N" (for null call), extract and reformat the sequence names, length and coordinates of the sites.

```
# Identify the first column containing sequence data for the samples
outgroup = which(varHeader=="X-3-7")
# Get the genome names
seqLabels = varHeader[outgroup:length(varHeader)]
# Re-format sequence names so sampling dates can be read by Path-O-Gen in Step 2
newSeqLabels = sapply(seqLabels,function(s) {
  s = gsub("a","-a",s) # Replace a suffixes with -a
  s = gsub("b","-b",s) # Replace b suffixes with -b
  s = gsub("c","-c",s) # Replace c suffixes with -c
  s = gsub("d","-d",s) # Replace d suffixes with -d
  s = gsub("-$","",s,fixed=TRUE) # Remove bloodstream indicators
  patientVec = unlist(strsplit(s,"-")) # Vector of patient, year, month, a/b/c/d
  print(patientVec)
  # Suffix isolates with an "a" if not specified
  # (i.e. if isolate represents the only sample from a patient)
  if(length(patientVec)==3){
    patientVec = c(patientVec,"a")
  }
  # New sequence name format: patient+sample_year
  sampleDate = format(round(as.numeric(patientVec[3])/12+as.numeric(patientVec[2]),
                            digits = 3), nsmall = 3)
  paste0(patientVec[1],patientVec[4],"_",sampleDate)
})
```

```
## [1] "X" "3" "7"
## [1] "A" "0" "0"
## [1] "A" "4" "2" "a"
## [1] "A" "4" "2" "b"
## [1] "A" "5" "0" "a"
## [1] "A" "5" "0" "b"
## [1] "B"  "4"  "11"
## [1] "B" "7" "1"
## [1] "B" "9" "1"
## [1] "B"  "11" "1"
## [1] "C"  "4"  "11"
## [1] "C" "8" "0"
```

```
## [1] "C"  "10" "0"
## [1] "C"  "12" "1"
## [1] "D"  "7"  "10"
## [1] "E" "6" "1"
## [1] "E" "8" "4"
## [1] "E" "9" "5"
## [1] "E"  "12" "1"
## [1] "E"  "13" "0"
## [1] "F" "6" "9"
## [1] "F"  "6"  "11"
## [1] "F" "7" "0" "a"
## [1] "G" "7" "5"
## [1] "G" "9" "8"
## [1] "G"  "9"  "11" "b"
## [1] "G"  "10" "3"
## [1] "G"  "10" "6"
## [1] "G"  "9"  "11" "a"
## [1] "G"  "9"  "11" "c"
## [1] "G"  "10" "0"  "a"
## [1] "G"  "10" "0"  "b"
## [1] "G"  "10" "0"  "c"
## [1] "G"  "10" "1"  "b"
## [1] "G"  "10" "2"
## [1] "G"  "10" "1"  "c"
## [1] "G"  "10" "11" "b"
## [1] "H" "8" "3"
## [1] "H" "9" "5"
## [1] "H"  "10" "1"
## [1] "H"  "10" "6"  "a"
## [1] "H"  "10" "6"  "b"
## [1] "H"  "9"  "10"
## [1] "H"  "9"  "11"
## [1] "H"  "10" "10"
## [1] "H"  "11" "3"
## [1] "H"  "12" "7"
## [1] "H"  "12" "10"
## [1] "H"  "12" "11" "a"
## [1] "H"  "13" "0"  "b"
## [1] "I" "8" "7"
## [1] "I" "9" "6"
## [1] "J"  "9"  "11"
## [1] "J"  "11" "8"
## [1] "J"  "11" "11"
## [1] "J"  "12" "4"
## [1] "J"  "12" "7"
## [1] "J"  "12" "11"
## [1] "J"  "13" "6"  "a"
## [1] "J"  "13" "6"  "b"
## [1] "J"  "14" "2"
## [1] "J"  "14" "8"
## [1] "K" "9" "0"
## [1] "K"  "10" "0"
## [1] "K"  "10" "1"
## [1] "K"  "10" "7"
```

```
## [1] "K"  "10" "2"
## [1] "K"  "10" "3"  "a"
## [1] "K"  "10" "10"
## [1] "K"  "11" "6"
## [1] "K"  "11" "3"  "a"
## [1] "K"  "11" "3"  "b"
## [1] "K"  "12" "5"
## [1] "K"  "12" "8"  "a"
## [1] "K"  "12" "0"  "a"
## [1] "K"  "12" "0"  "b"
## [1] "K"  "12" "9"
## [1] "L"  "9"  "11"
## [1] "L"  "10" "1"
## [1] "L"  "10" "2"
## [1] "L"  "10" "6"
## [1] "L"  "10" "10"
## [1] "L"  "11" "3"
## [1] "L"  "11" "6"
## [1] "L"  "11" "8"
## [1] "L"  "11" "11"
## [1] "L"  "12" "3"
## [1] "L"  "12" "7"
## [1] "L"  "13" "0"
## [1] "L"  "13" "7"
## [1] "L"  "13" "4"  "a"
## [1] "L"  "16" "0"
## [1] "M" "9" "4"
## [1] "M"  "10" "6"
## [1] "M"  "11" "5"
## [1] "M"  "12" "6"
## [1] "N"  "10" "0"
## [1] "N"  "10" "1"
## [1] "N"  "10" "5"
## [1] "N"  "10" "8"
## [1] "N"  "10" "11"
## [1] "N"  "11" "2"
## [1] "N"  "11" "3"
## [1] "N"  "11" "7"
## [1] "N"  "11" "9"
## [1] "N"  "12" "1"
## [1] "N"  "12" "6"  "a"
## [1] "N"  "12" "6"  "c"
## [1] "N"  "12" "6"  "d"
## [1] "N"  "12" "6"  "b"
## [1] "N"  "12" "4"  "a"
## [1] "N"  "12" "5"
## [1] "N"  "12" "4"  "c"
```

```r
# Count the number of genomes
nSeq = length(seqLabels)
# Extract base calls & transpose so each row is a genome
vSites = t(variants[,outgroup:ncol(variants)])
# Ensure all characters are in upper case
vSites = toupper(vSites)
```

```r
# Replace ambiguous calls
vSites[!(vSites=="A" | vSites=="C" | vSites=="G" | vSites=="T")] = "N"
# Get the total length of the reference genome
lengthRef = length(AU0158)
# Convert the chromosome::position coordinates to concatenated coordinates
vPos = sapply(variants[,1],function(s) {
  tp = as.numeric(unlist(strsplit(s,"::")))
  c(0,cumsum(chromLen))[tp[1]]+tp[2]
})
# Sanity check: make sure the reference base in Lieberman_etal_variants.tab.txt
# matches the reference base in B.dolosa.AU0158.fa.
# It's good practice to include sanity checks into analyses
table(variants[,6],AU0158[vPos])
```

```
##
##       A   C   G   T
##   A  95   0   0   0
##   C   0 167   0   0
##   G   0   0 158   0
##   T   0   0   0  91
```

Preparation of nucleotide sequence data into the correct format is an important initial step in any population genetics analysis and different programs can require different input formats (FASTA, NEXUS, PHYLIP, Clustal, GenBank). Once a multiple sequence alignment has been obtained from a bioinformatics pipeline, it may require conversion to a different file type. We will be reconstructing the phylogenetic tree using maximum likelihood with the program PhyML, which reads in a sequence alignment in PHYLIP format. Sequentially write a PHYLIP format file containing the sequence names and the full alignment, including invariant sites. This file will require around 720MB of disk space.

```r
# Specify PHYLIP file name
phylipOutfile = "Lieberman_etal_alignment.phylip"
# Output PHYLIP header: sequence number and sequence length
cat(paste(nSeq,lengthRef),sep="\n",file=phylipOutfile)
# For each genome, append to the PHYLIP file
for(i in 1:nSeq) {
  # Begin by setting every site to that of the reference
    fullLengthRef = AU0158
  # Then replace the genome-specific calls at variable sites
    fullLengthRef[vPos] = vSites[i,]
  # Concatenate the sequence vector and output
    fullLengthRefCat = paste0(fullLengthRef,collapse="")
    cat(paste(newSeqLabels[i]," ",fullLengthRefCat,sep=""),
      sep="\n",file=phylipOutfile,append=TRUE)
    cat("Output",i,"of",nSeq,"\n")
}
```

We will also require the sequence data in FASTA format for input into FastML in Step 4. This step only requires the variant sites to be written to file (so it only requires around 60KB of disk space).

```r
fastaOutfile = "Lieberman_etal_alignment_varSites.fasta" # Specify FASTA file name
# For each genome, append to the FASTA file
for(i in 1:nSeq) {
  cat(paste(">", newSeqLabels[i], sep=""),sep="\n",file=fastaOutfile,append=TRUE)
```

```
 seq = paste0(vSites[i,], collapse="")
 cat(seq, sep="\n", file=fastaOutfile,append=TRUE)
}
```

STEP 1: Construction of a phylogenetic tree

For principled statistical inference of phylogenetic trees, an explicit model of sequence evolution is required. Software for phylogenetic inference usually offers a great deal of flexibility in these models. In the following, a phylogeny for the *B. dolosa* isolates is estimated by maximum likelihood using the PhyML software, under the HKY85 substitution model, and a single substitution rate for all sites (2). At the command line, execute:

```
phyml -i Lieberman_etal_alignment.phylip -b 0 -v 0 -c 1 -s BEST --no_memory_check
```

The options specify the following: -i input file, -b number of bootstrap replicates (none), -v proportion of sites in the genome that cannot mutate due to inviability selection (none), -c number of different substitution rate classes (one), -s tree exploration strategy, –no_memory_check disable a prompt asking about memory usage for the software. To find out more, type phyml –help.

Estimating the phylogeny takes a couple of minutes and produces two files:

- Lieberman_etal_alignment.phylip_phyml_tree.txt (containing the tree in Newick format)
- Lieberman_etal_alignment.phylip_phyml_stats.txt (summarizing the results).

Look in the second file, which contains information about the run and the results. You will see that we specified an HKY85 model of nucleotide substitution. While some substitution models specify equal base frequencies or rates of transition between bases, the Hasegawa-Kishino-Yano 1985 (HKY85) model accounts for differences in base frequencies (f) and between the rate of transitions and transversions, modeled with the parameter κ. You should see further down in the file that the value of κ has been estimated by PhyML to be 2.222 for this dataset (we will need this value this later on for use in a codon substitution model).

A wide range of software is available for visualizing phylogenies. FigTree is an intuitive graphical user interface that makes it easy to quickly produce an attractive figure. The file can be loaded into FigTree by clicking File -> Open and then selecting the file Lieberman_etal_alignment.phylip_phyml_tree.txt. The phylogeny can also be opened in FigTree from the command line:

```
figtree Lieberman_etal_alignment.phylip_phyml_tree.txt
```

To interpret the tree, consider the relationships between samples from the same individual and different individuals (genomes are labeled alphabetically A-N representing the patient from whom they were sampled). Remember that the tree may need to be rooted before you can interpret the direction of evolution (i.e. distinguish ancestral versus derived/mutant alleles). In FigTree, it is possible to manually color-code branches of the tree to make the patient-of-origin clearer, and the tree can be midpoint rooted. Try experimenting with the layout to improve the figure.

It is also possible to plot the tree in R using color-coding of the patients. This requires the use of an R package called ape, which includes a variety of phylogenetic based methods. R packages can be installed using the following code from within R, for example:

```
install.packages(ape)
```

Further details on installing R packages can be found online: http://cran.r-project.org/doc/manuals/r-release/R-admin.html#Installing-packages).

```r
library(ape)
# Load the ML tree
MLTree = read.tree("Lieberman_etal_alignment.phylip_phyml_tree.txt")
# Re-root the tree on the taxon representing the index case
MLTree = root(MLTree, "Aa_0.000")
# Get the patient ID from the tip labels
patientLetters = sapply(MLTree$tip.label, function(t){
  substring(t, 1, 1)
})
# Convert patient letters to integers
patientInts = match(matrix(patientLetters), levels(as.factor(patientLetters)))
# Assign color to each patient according to patient integers
cols = sapply(patientInts, function(c){
  rainbow(max(patientInts))[c]
  })
# Optional: ladderize the tree to improve it's appearance (only affects
# node rotations)
MLTree = ladderize(MLTree)
# Plot the tree
plot(MLTree, type = "phylo", show.tip.label = TRUE, tip.col = cols, cex = 0.5,
     edge.width = 2)
# Add a legend for the tip colors
legend("bottomleft",
       c(LETTERS[seq( from = 1, to = length(unique(patientInts))-1)],"X (outgroup)"),
       col = rainbow(max(patientInts)), lty = 1, lwd = 4, cex = 1,
       title = "Patient letters")
```

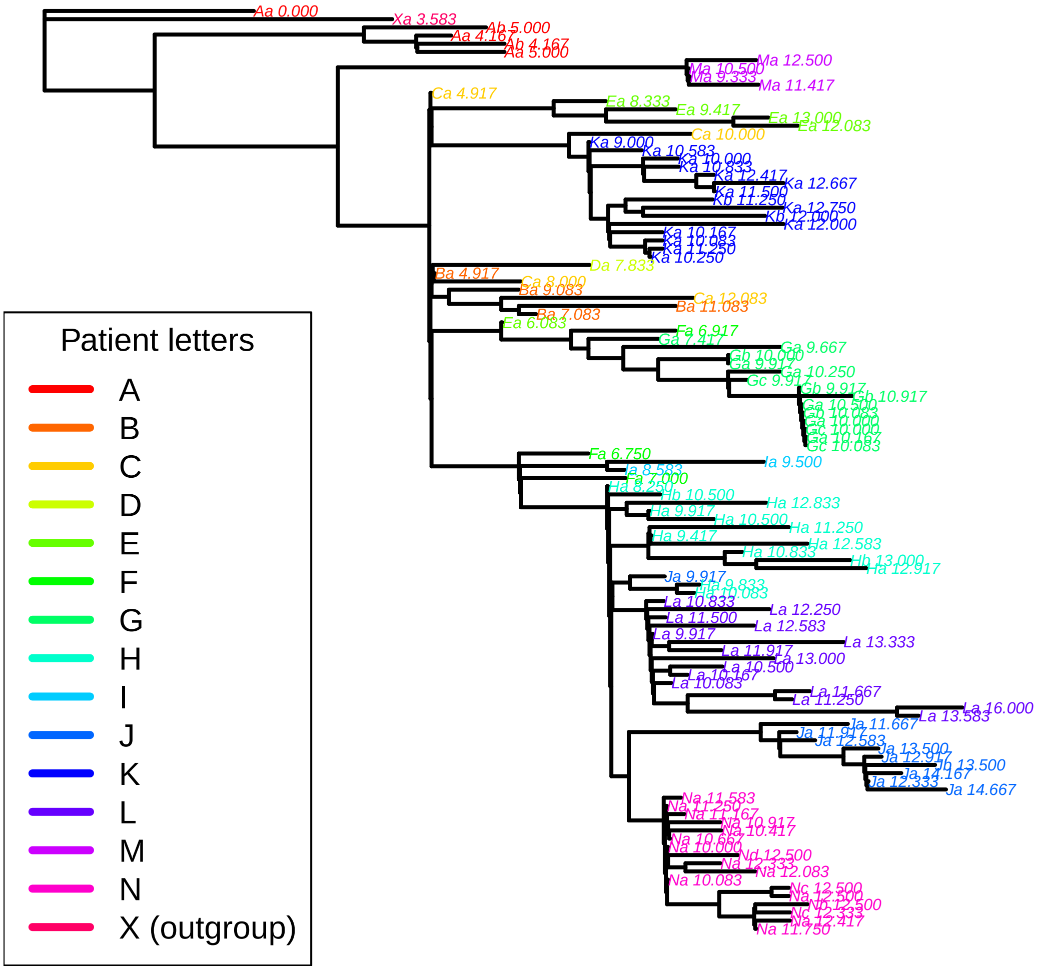

STEP 2: Estimation of the evolutionary rate

Without calibrating the rate of molecular evolution, the timescale of a phylogeny cannot be interpreted. If the tree is rooted on a suitably divergent outgroup sequence and the sampling interval needs to have been sufficiently wide for substitutions to accumulate, it's possible to test for a temporal signal in the data by performing a linear regression. This regresses the sampling time of each sequence versus the total branch length between its position in the tree from the root. Here, we will use the sequence "Xa_3.583" as an outgroup to root the tree, which was sampled from a patient infected with *B. dolosa* independently of the Boston outbreak.

```
# First, load the phytools library
library(phytools)
```

```
## Loading required package: maps
```

```
# Root the tree with the outgroup taxon
MLTree = root(MLTree, outgroup = "Xa_3.583")
# Get the distance from the root for every branch using the nodeHeights function
# which is part of the phytools library
rootDists = cbind(nodeHeights(MLTree)[,2],MLTree$edge[,2])
# Select only those branches which are tips
```

```r
rootDists = rootDists[which(rootDists[,2] <= length(MLTree$tip.label)),]
# Get the tip label corresponding to every tip (in the same order as rootDists)
orderedTipLabs = MLTree$tip.label[rootDists[,2]]
# Add the tip label to the table of distances from the root
rootDists = cbind(rootDists, orderedTipLabs)
# Get the decimalized sample time for each sequence from its name (separated by "_")
times = sapply(rootDists[,3], function(x){
  decDate = matrix(unlist(strsplit(x, "_")))
    return(as.numeric(decDate[2,]))
    })
# Convert the distances in the rootDists table to numbers
distances = as.numeric(rootDists[,1])
# Fit a linear model to the data to estimate the slope (evolutionary rate)
# and intercept (date of the most recent common ancestor)
fit = lm(distances~times)
fit_summary = summary(fit)$fstatistic
# Calculate the p-value, intercept and slope
p = pf(fit_summary[1],fit_summary[2],fit_summary[3],lower.tail=F)
intercept = (-fit$coefficients[1])/fit$coefficients[2]
slope = fit$coefficients[2]
# Plot the data
plot(times, distances, pch = 16,
     xlab = "Time since the first sample (years)",
     ylab = "Distance from root (substitutions/site)", ylim = range(0, max(distances)),
     xlim = range(intercept, max(times)))
# Plot the regression line
abline(fit, col="red", lwd = 2)
# Add the R-squared statistic, p-value and slope
r_squared = format(summary(fit)$adj.r.squared, digits=4)
p_value = format(p, digits = 4)
slope_value = format(slope, digits = 4)
text(x = 0, y = 1e-6, bquote(R^2 ~ " = " ~ .(r_squared)), p = 4)
text(x = 0, y = 5e-7, bquote("P-value = " ~ .(labels = p_value)), p = 4)
text(x = 0, y = 1e-7, bquote("Slope = " ~ .(labels = slope_value)), p = 4)
```

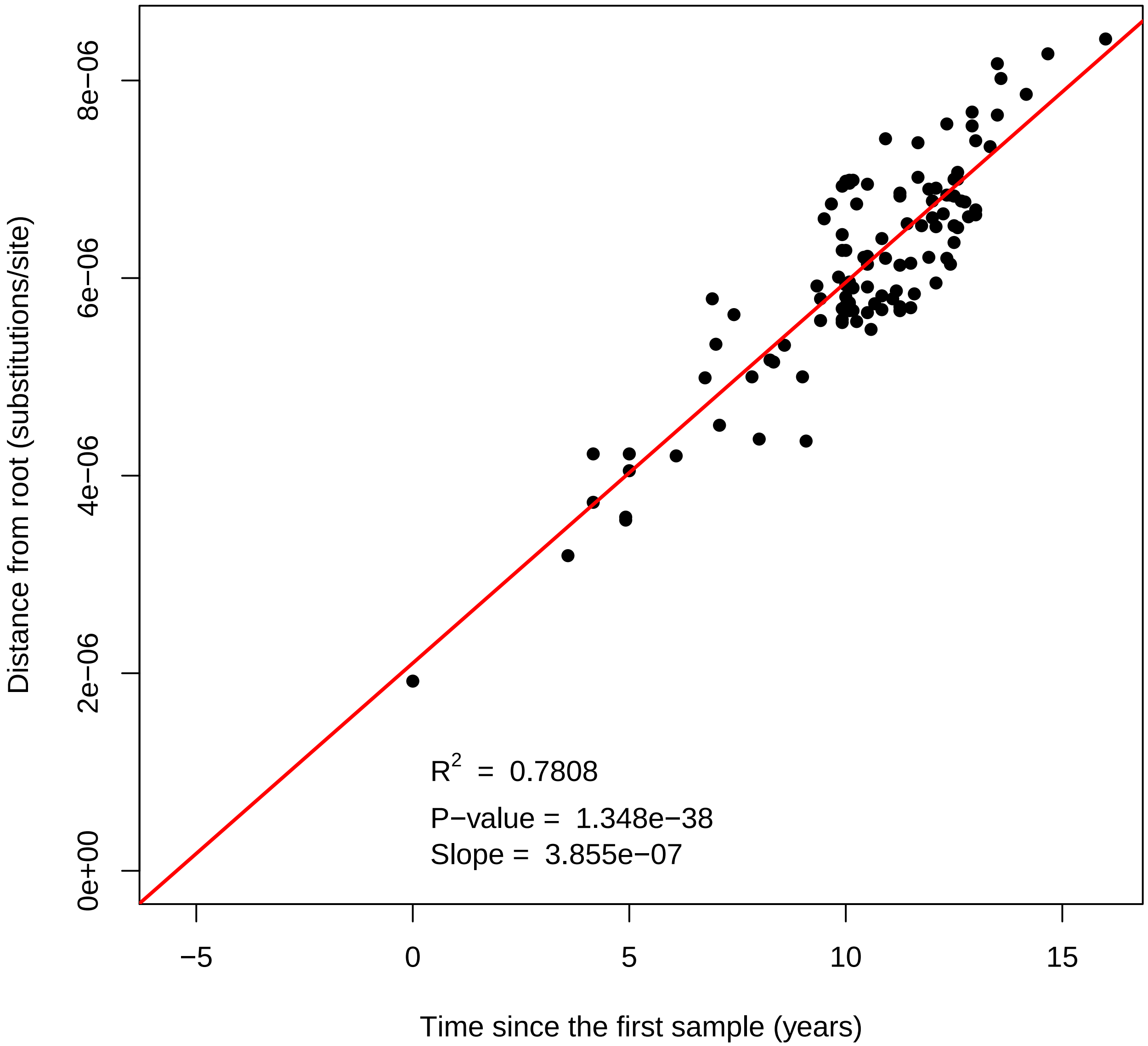

In the absence of an obvious outgroup, the software Path-O-Gen can be used to perform a similar analysis by rooting the tree at the position most compatible with the data evolving according to a molecular clock (http://tree.bio.ed.ac.uk/software/pathogen/).

Opening Path-O-Gen and load the relabeled tree by clicking File -> Open and selecting the tree file. The Path-O-Gen tool can guess the sampling dates from the genome names, so check that dates are specified as "Years" and "Since some time in the past" and then select "Guess Dates". In the window that opens, check the first option "Defined just by its order", choose "last" from the pull down "Order" menu and then click OK. Click on the "Analysis" button and check the "Best-fitting root" box.

The panel on the left gives an estimate of the molecular clock rate ("Slope (rate)") in units of substitutions per site per year. To obtain a per-genome rate, multiply by the length of the genome (6,420,400 base pairs). This should provide an estimate of around 2.40 substitutions per genome per year. The X-intercept also provides an estimate of the time of the most recent common ancestor (TMRCA), provided here in years before the time of the first sample (around 5 years). The "Tree" and "Root-to-tip" tabs offer different ways to visualize the molecular clock signal. Notice that the best-fitting root, which takes into account the sampling dates, is not the same as the midpoint root.

STEP 3: Genome annotation

The xBASE annotation service (http://www.xbase.ac.uk/annotation/) is a fast and convenient online pipeline for genome annotation. You can use it to annotate the AU0158 reference genome, which will provide a starting point for classifying substitutions.

Navigate to the website and click on the "Select Reference Genome" button. Type "Burkholderia" and choose one of the well-annotated high-quality reference genomes in the xBASE database. Note that *B. dolosa* is rare and a complete reference doesn't currently exist in the xBASE database. For example, you could choose *Burkholderia cenocepacia* AU1054.

Next, enter your email address and upload the FASTA file called B.dolosa.AU0158.fa, which contains the AU0158 reference genome. Enter appropriate values for the "Species name" and "Strain identified" and then click the "Submit Sequence for Annotation" button.

It takes around 12 minutes for the annotation to complete. When your own annotation is complete, you will get an email with an HTML link to the annotated genome. If you wish to check your output with the existing copy, download the "Concatenated annotation in GenBank format (good for Artemis/ACT)" file and save it as "B.dolosa.AU0158.xbase.gbk". You can browse the annotation in Artemis (3). At the command line type:

```
art B.dolosa.AU0158.xbase.gbk
```

Click "OK" and ignore the warning messages. If you go to the "View" menu and select "CDS Genes and Products" you can quickly scroll through the gene annotations. Use "Ctrl G" to go to a specific position in the genome.

STEP 4: Classification of substitutions

In order to count all synonymous, non-synonymous, nonsense or intergenic substitutions, ancestral sequence reconstruction is required. This can be performed using maximum likelihood in FastML, which can either be run on a web server (http://fastml.tau.ac.il/) or from the command line using a Linux operating system if the software is installed locally (4). First, make a new folder in your current working directory for the output of FastML from the command line:

```
mkdir FastML_output
```

Running FastML on either the webserver or from the command line requires specification of the name of the FASTA file (here, we are just using the variant sites for computational efficiency), the data type (nucleotide) and the tree (ML tree produced earlier using PhyML). The command line version also needs the name of the output directory we just created (including the full path) and can be run using the following command:

```
perl FastML.v3.1/www/fastml/FastML_Wrapper.pl --MSA_File Lieberman_etal_alig
nment_varSites.fasta --outDir ~/Documents/S1_file/FastML_output --seqType nuc
--Tree Lieberman_etal_alignment.phylip_phyml_tree.txt
```

FastML outputs six files, including a two FASTA files of extant and ancestral sequences from the joint reconstruction (sequences are reconstructed simultaneously, by finding the most likely set of sequences across all nodes) or marginal reconstruction (each sequence is estimated separately by averaging over all possible sequences at other nodes). These are saved in seq.joint.txt and seq.marginal.txt respectively and the input Newick tree annotated with node labels is saved in tree.newick.txt. By comparing sequences at either end of each branch in the tree, the total number of non-synonymous and synonymous substitutions occurring over the evolutionary history of this sample can be counted. This count will underestimate the true number of substitutions that have occurred since this method assumes that only one change per site occurs along each branch of the tree.

First, load in two additional R libraries containing functions for reading GenBank files and performing sequence analysis.

```
library("genoPlotR")
library("seqinr")
library("geiger")
```

We can then read in the GenBank file from xBASE and the output files from FastML (or use the copy in annotationOutput).

```
gbk = read_dna_seg_from_file("B.dolosa.AU0158.xbase.gbk", fileType="genbank",
                             extra_fields="*matching_locus_tag")
# Read in imputed sequence data from the joint reconstruction and the Newick tree
# (internal nodes are labeled "N" followed by a number allocated by FastML)
MLSeqFile=scan("FastML_output/seq.joint.txt",what=character(0))
# Store sequences in vector
MLSeqs = MLSeqFile[seq(2,length(MLSeqFile),by=2)]
fastMLTree = read.tree("FastML_output/tree.newick.txt")
```

In order to identify the reconstructed sequences corresponding to each node in the tree, we can store the sequences in a matrix.

```
# Initialize empty matrix, M, in which sequences will be stored
M = matrix("",length(MLSeqs),nchar(MLSeqs[1]))
# Fill each row of M, where each column is a variant site and
# each row is a sequence
for(i in 1:length(MLSeqs)) {
  seq = as.matrix(unlist(strsplit(MLSeqs[i], "")))
  M[i,] = seq
}
# Store all sequence names in vector, removing > at start of each name
seqLabels = gsub(">", "", MLSeqFile[seq(1,length(MLSeqFile), by = 2)])
rownames(M) = seqLabels # Assign sequence names to sequence vector
```

Using the ape library, we can match the sequence at each node or tip with the sequence at its parent node. These relationships were calculated when the tree was read in by the ape library and are stored as a matrix which can be accessed by typing fastMLTree$edge.

```
# Get all internal node and tip labels
nodeLabels = c(fastMLTree$tip.label,fastMLTree$node.label)
# Reorder M so that sequences are in the same order as nodeLabels
M = M[match(nodeLabels, rownames(M)),]
# For each node, get the row index of its ancestor
M_ancestorNodeIndex = sapply(1:nrow(M), function(x){
  edgeRowNumber = match(x, fastMLTree$edge[,2])
  return(fastMLTree$edge[edgeRowNumber,1])
})
```

Next, provide the genetic code for translating codons into amino acids and a vector for reverse complementing negative strand DNA sequences.

```
geneticCode = list(
"TTT"="Phe","TTC"="Phe","TTA"="Leu","TTG"="Leu",
"TCT"="Ser","TCC"="Ser","TCA"="Ser","TCG"="Ser",
"TAT"="Tyr","TAC"="Tyr","TAA"="STO","TAG"="STO",
"TGT"="Cys","TGC"="Cys","TGA"="STO","TGG"="Trp",
"CTT"="Leu","CTC"="Leu","CTA"="Leu","CTG"="Leu",
"CCT"="Pro","CCC"="Pro","CCA"="Pro","CCG"="Pro",
"CAT"="His","CAC"="His","CAA"="Gln","CAG"="Gln",
"CGT"="Arg","CGC"="Arg","CGA"="Arg","CGG"="Arg",
"ATT"="Ile","ATC"="Ile","ATA"="Ile","ATG"="Met",
"ACT"="Thr","ACC"="Thr","ACA"="Thr","ACG"="Thr",
"AAT"="Asn","AAC"="Asn","AAA"="Lys","AAG"="Lys",
"AGT"="Ser","AGC"="Ser","AGA"="Arg","AGG"="Arg",
"GTT"="Val","GTC"="Val","GTA"="Val","GTG"="Val",
"GCT"="Ala","GCC"="Ala","GCA"="Ala","GCG"="Ala",
"GAT"="Asp","GAC"="Asp","GAA"="Glu","GAG"="Glu",
"GGT"="Gly","GGC"="Gly","GGA"="Gly","GGG"="Gly")

complement = c("A"="T","C"="G","G"="C","T"="A","N"="N")
```

Then calculate the number of alleles at each variable site.

```
nNuc = apply(vSites,2,function(x)table(factor(x,levels=c("A","C","G","T"))))
nAlleles = colSums(nNuc>0)
```

We also need to write some specialized functions that are not provided by R. The first function reads in the index (i) of each variant site in the vector vPos and the number of alleles at site i. It returns a vector of information about substitutions at that site.

```
subType = function(i, numberOfAlleles){
  # Set all counts of substitution types to zero
    nonSynCount = 0
    synCount = 0
    readThroughCount = 0
    nonsenseCount = 0
    position = as.numeric(vPos[i])
  # We will fill in the entries in codonInfo below
    codonInfo = c("Type"="", "NonSynCount"=0,"SynCount"=0,
                  "ReadThroughCount"=0, "NonsenseCount"=0, "Refcodon"="",
                  "Nonrefcodon"="-","Frame"="-","Codonposition"="-",
                  "Refaa"="-","Nonrefaa"="-")
    refAllele = AU0158[position]
    if(is.na(position)) {
        codonInfo[1] = "Unknown"
        return(codonInfo)
    }
    geneIndex = which((gbk$feature=="CDS" & gbk$start<=position & gbk$end>=position))
    if(length(geneIndex)==0) {
        codonInfo[1] = "Intergenic"
        return(codonInfo)
    }
    if(numberOfAlleles==1){
        codonInfo[1] = "Invariant"
```

```
    return(codonInfo)
}
# Find reading frame of gene and indices of all 3 sites in codon
if(gbk$strand[geneIndex]==1) { # Codon is on the positive strand
    frame = (position-gbk$start[geneIndex]) %% 3
    codonPositions = position-frame+(0:2)
    refCodon = paste(AU0158[codonPositions],collapse="")
}else{ # Codon is on the negative strand and requires reverse complementation
    frame = (gbk$end[geneIndex]-position) %% 3
    codonPositions = position+frame-(0:2)
    refCodon = paste(complement[AU0158[codonPositions]],collapse="")
}
for(j in 1:nrow(M)){ # For each branch in tree
# Get node and ancestral bases
nodeVarBase = M[j, i]
ancestralNodeNumber = M_ancestorNodeIndex[j]
ancestorVarBase = M[ancestralNodeNumber, i]
# If not root and bases are different
if(!is.na(ancestorVarBase) && nodeVarBase != ancestorVarBase){
  nodeCodon = AU0158[codonPositions] # Set node codon to reference
  ancestorCodon = AU0158[codonPositions] # Set ancestral codon to reference
  for(x in c(1, 2, 3)){
    if(codonPositions[x] %in% vPos){
      # Get column index in matrix M
      M_columnIndex = match(codonPositions[x], vPos)
      # Substitute codon position in node and ancestral codons for correct bases
      nodeBase = M[j, M_columnIndex]
      nodeCodon[x] = nodeBase
      ancestralBase = M[ancestralNodeNumber, M_columnIndex]
      ancestorCodon[x] = ancestralBase
    }
    }
  if(gbk$strand[geneIndex]==-1) { # If negative strand, complement bases
    nodeCodon = as.vector(complement[nodeCodon])
    ancestorCodon = as.vector(complement[ancestorCodon])
  }
  # If "N" in either codon, we can't count any substitutions
  if(!"N" %in% nodeCodon && !"N" %in% ancestorCodon){
    nodeCodonCol = paste0(nodeCodon, collapse="")
    ancestorCodonCol = paste0(ancestorCodon, collapse="")
        nodeAA = as.character(geneticCode[nodeCodonCol])
        ancestralAA = as.character(geneticCode[ancestorCodonCol])
        if(nodeAA == ancestralAA){
            synCount = synCount + 1
        }else if(ancestralAA == "STO"){
            readThroughCount = readThroughCount + 1
        }else if(nodeAA == "STO") {
            nonsenseCount = nonsenseCount + 1
        }else{
            nonSynCount = nonSynCount + 1
        }
  }
}
```

```
  }
    codonInfo = c("Type"="Coding", "NonSynCount"=nonSynCount,
                  "SynCount"=synCount,"ReadThroughCount"=readThroughCount,
                  "NonsenseCount"=nonsenseCount, "Refcodon"=refCodon,
                  "Nonrefcodon"="-","Frame"=frame+1,
                  "Codonposition"=((position-gbk$start[geneIndex]) %/% 3)+1,
                  "Refaa"="-","Nonrefaa"="-")
    return(codonInfo)
}
```

We also need a function to return the gene names(s) within which a site lies.

```
gbkLocate = function(gbk,position) {
  position = as.numeric(position)
    geneIndex = which(gbk$start<=position & position<=gbk$end)
    start = gbk$start[geneIndex]
    end = gbk$end[geneIndex]
    noMatch = length(start)==0 | length(end)==0
    if(!noMatch){
        noMatch = noMatch | is.na(start) | is.na(end)
    }
    if(noMatch) {
        tp = gbk[1,]
        tp[1,1:ncol(tp)] = "-"
        return(tp)
    }
    as.data.frame(t(apply(gbk[geneIndex,],2,paste0,collapse=":")))
}
```

Finally, using the functions above, construct a table called “subAnnotation”, which contains counts of non-synonymous and synonymous substitutions for each variant site, in addition to information about the site:

```
subAnnotation = list()
for(i in 1:length(vPos)){
  alleles = c("A","C","G","T")[order(nNuc[,i])][c(4,3)]
    siteInfo = data.frame("Position"=vPos[i],"MajorAllele"=alleles[1],
                          "MinorAllele"=alleles[2], "NumberOfAlleles"=nAlleles[i],
                          stringsAsFactors = FALSE)
    subInfo = t(subType(i, siteInfo$NumberOfAlleles))
    geneInfo = gbkLocate(gbk,siteInfo$Position[1])[,c("name","start","end","strand",
                                                       "length","pid","gene","synonym",
                                                       "product","proteinid","feature",
                                                       "*matching_locus_tag")]
  # Take the first gene if site in multiple overlapping genes
    subAnnotation = rbind(subAnnotation,
                          cbind(siteInfo[1,], subInfo, geneInfo[1,]))
}

subAnnotation = transform(subAnnotation,
                          NonSynCount = as.numeric(as.character(NonSynCount)),
                          SynCount = as.numeric(as.character(SynCount)))
table(subAnnotation$Type) # Summarize classifications
```

```
##
##     Coding Intergenic
##        428         83
```

```r
head(subAnnotation) # Preview the results
```

```
##           Position MajorAllele MinorAllele NumberOfAlleles       Type
## 1::5666       5666           C           G               2     Coding
## 1::8477       8477           G           T               2     Coding
## 1::22647     22647           C           G               2     Coding
## 1::23774     23774           A           C               2     Coding
## 1::26068     26068           A           G               2 Intergenic
## 1::51329     51329           G           T               2     Coding
##           NonSynCount SynCount ReadThroughCount NonsenseCount Refcodon
## 1::5666             1        0                0             0      CGC
## 1::8477             0        1                0             0      GGG
## 1::22647            1        0                0             0      GCG
## 1::23774            1        0                0             0      CAA
## 1::26068            0        0                0             0
## 1::51329            1        0                0             0      GCG
##           Nonrefcodon Frame Codonposition Refaa Nonrefaa        name start
## 1::5666             -     1           285     -        - AU0158_0009  4814
## 1::8477             -     3            68     -        - AU0158_0012  8274
## 1::22647            -     2           140     -        - AU0158_0023 22229
## 1::23774            -     2           195     -        - AU0158_0024 23191
## 1::26068            -     -             -     -        -           -     -
## 1::51329            -     2            78     -        - AU0158_0058 51097
##             end strand length pid gene     synonym
## 1::5666    5902      1    362  NA    - AU0158_0009
## 1::8477    8921      1    215  NA    - AU0158_0012
## 1::22647 23194      1    321  NA    - AU0158_0023
## 1::23774 23838      1    215  NA    - AU0158_0024
## 1::26068     -      -      -   -    -           -
## 1::51329 51441     -1    114  NA    - AU0158_0058
##                                                    product proteinid
## 1::5666  phenylacetate-CoA oxygenase/reductase, PaaK subunit        NA
## 1::8477                      GCN5-related N-acetyltransferase        NA
## 1::22647                                 hypothetical protein        NA
## 1::23774                                                   NA        NA
## 1::26068                                                    -         -
## 1::51329      histone-like nucleoid-structuring protein H-NS        NA
##           feature X.matching_locus_tag
## 1::5666       CDS                   NA
## 1::8477       CDS                   NA
## 1::22647      CDS                   NA
## 1::23774      CDS                   NA
## 1::26068        -                    -
## 1::51329      CDS                   NA
```

```r
# Compare top 30 gene annotations to Lieberman et al
head(cbind(variants[,4],as.character(subAnnotation$product)),n=30)
```

```
##       [,1]
```

```
##  [1,] "Flavodoxin reductase"
##  [2,] "Histone acetyltransferase HPA6"
##  [3,] ""
##  [4,] ""
##  [5,] ""
##  [6,] "H-NS histone family protein"
##  [7,] "Methyl coenzyme M reductase alpha subunit"
##  [8,] "hypothetical protein"
##  [9,] "hypothetical protein"
## [10,] "hypothetical protein"
## [11,] "Type II secretory pathway component PulD"
## [12,] "Type II secretory pathway component PulD"
## [13,] "DNA-directed RNA polymerase specialized sigma subunit"
## [14,] ""
## [15,] "Type IIA topoisomerase"
## [16,] "Type IIA topoisomerase"
## [17,] "Type IIA topoisomerase"
## [18,] "hypothetical protein"
## [19,] ""
## [20,] "; Multi-sensor signal transduction histidine kinase; frameshift"
## [21,] "P pilus assembly protein porin PapC"
## [22,] "hypothetical protein"
## [23,] "Rhs family protein"
## [24,] "Rhs family protein"
## [25,] "; Sensor histidine kinase; frameshift"
## [26,] "; Sensor histidine kinase; frameshift"
## [27,] "Lauroyl/myristoyl acyltransferase"
## [28,] "Pyruvate/2-oxoglutarate dehydrogenase complex dihydrolipoamide dehydrogenase (E3) component"
## [29,] "Spermidine/putrescine-binding periplasmic protein"
## [30,] "Conserved hypothetical protein"
##       [,2]
##  [1,] "phenylacetate-CoA oxygenase/reductase, PaaK subunit"
##  [2,] "GCN5-related N-acetyltransferase"
##  [3,] "hypothetical protein"
##  [4,] "NA"
##  [5,] "-"
##  [6,] "histone-like nucleoid-structuring protein H-NS"
##  [7,] "hypothetical protein"
##  [8,] "NA"
##  [9,] "NA"
## [10,] "-"
## [11,] "Type II secretion system gspD"
## [12,] "Type II secretion system gspD"
## [13,] "RNA polymerase sigma factor SigJ"
## [14,] "-"
## [15,] "DNA gyrase subunit B"
## [16,] "DNA gyrase subunit B"
## [17,] "DNA gyrase subunit B"
## [18,] "-"
## [19,] "DNA processing protein DprA, putative"
## [20,] "periplasmic sensor signal transduction histidine kinase"
## [21,] "fimbrial biogenesis outer membrane usher protein"
## [22,] "OmpA/MotB"
## [23,] "PAAR"
```

```
## [24,] "PAAR"
## [25,] "periplasmic sensor signal transduction histidine kinase"
## [26,] "periplasmic sensor signal transduction histidine kinase"
## [27,] "lipid A biosynthesis lauroyl acyltransferase"
## [28,] "glutathione reductase"
## [29,] "extracellular solute-binding protein"
## [30,] "putative aminotransferase"
```

Not all of the variants are found in genes that have been annotated by xBASE. Since the annotation of the reference sequence we used was incomplete, further insights into the functional significance of these variants might be gained from querying these sequences with BLAST (https://blast.ncbi.nlm.nih.gov/Blast.cgi). This online server searches for the most closely related sequences from a huge database of annotated nucleotide sequences and might provide some clues regarding the type of sequence in which these variants are located.

Now that we've counted and classified all substitutions in the tree, we need to calculate empirical codon usage from the AU0158 reference genome before we can quantify the selection acting on the data:

```
concatTranscripts = c()
for(i in 1:nrow(gbk)) {
    if(gbk$strand[i]==1){
    concatTranscripts = c(concatTranscripts,apply(matrix(
      toupper(AU0158[gbk$start[i]:gbk$end[i]]),nrow=3),2,paste,collapse=""))
  }
  if(gbk$strand[i]==-1){
    concatTranscripts = c(concatTranscripts,apply(matrix(complement[
      toupper(AU0158[gbk$end[i]:gbk$start[i]])],nrow=3),2,paste,collapse=""))
  }
}
codonUsage = table(factor(concatTranscripts,levels=names(geneticCode)))
```

The following website can also be used to obtained tables of codon usage for a range of species: http://www.kazusa.or.jp/codon/cgi-bin/showcodon.cgi?species=331271. For example, by selecting the reference genome AU1054, you can obtain the table above that we have just calculated in R.

STEP 5: Testing for Selection

Step 5 is composed of parts A-C that use three different methods to estimate the selective pressure acting the *B. dolosa* genome sequences.

For all steps, we will need the list of gene names, which can be extracted from the GenBank annotation file:

```
geneNames = gbk$name
# Remove duplicate gene names
geneNames = geneNames[!duplicated(geneNames)]
# Lengths of genes
geneLengths = xtabs((gbk$end-gbk$start+1)~factor(gbk$name,levels=geneNames))
```

Using the list of information about each substitution created in Step 4 (called "subAnnotation"), count the number of synonymous and non-synonymous substitutions that occur in each gene.

```
# Count the number of non-synonymous and synonymous substitutions in each gene
rawNS = data.frame(matrix(0, nrow = length(geneNames), ncol = 2))
rownames(rawNS) = geneNames
```

```r
colnames(rawNS) = c("N", "S")
# Find all coding substitutions
codingSubs = subAnnotation[which(subAnnotation$Type == "Coding"),]
# Convert counts of non-synonymous and synonymous substitution to numeric objects
aggCounts = aggregate(codingSubs[,6:7],
                      list(as.character(codingSubs$name)), sum)
# Get counts across all genes (i.e. including invariant genes)
rawNS[match(aggCounts[,1], geneNames),] = aggCounts[,2:3]
```

Now we have all the information required to carry out various tests for selection.

A) Elevated substitution rates signal positive selection

It might be expected that a gene under positive selection is more likely to be mutated multiple times, where each mutation gives rise to a similar, advantageous change in phenotype. Conversely, a gene under negative selection is assumed to have fewer mutations than expected by chance. This first approach tests for differences in selection across genes by testing for the presence of a significantly different number of substitutions than expected in a gene, given the substitution rate estimate for the whole genome. This approach does not distinguish between different types of substitution.

```r
# Total number of non-synonymous and synonymous substitutions for each gene
nSubs = rawNS[,1] + rawNS[,2]
# Substitution rate per site
subRate = sum(nSubs)/sum(geneLengths)
# Calculate the p-value for each gene
subRatePVal = sapply(1:length(nSubs),function(i){
  t = poisson.test(nSubs[i],r=(subRate*geneLengths[i]))
  t$p.value
})
# Get the genes with significantly more substitutions than expected by chance
subRateSignif = geneNames[which(subRatePVal<0.01)]
(subRateSignif)
```

```
##  [1] "gyrB"        "AU0158_0754" "AU0158_1082" "AU0158_1458" "AU0158_1477"
##  [6] "AU0158_1478" "AU0158_1543" "AU0158_1582" "AU0158_1852" "AU0158_1913"
## [11] "AU0158_2053" "AU0158_2428" "AU0158_2535" "AU0158_2607" "AU0158_2802"
## [16] "AU0158_2931" "AU0158_2984" "AU0158_3038" "AU0158_3063" "AU0158_3143"
## [21] "AU0158_3222" "AU0158_3249" "AU0158_3327" "AU0158_3402" "infA"
## [26] "rplD"        "AU0158_3666" "AU0158_3855" "AU0158_4188" "AU0158_4512"
## [31] "AU0158_4835" "AU0158_5195" "AU0158_5441" "AU0158_5476" "AU0158_5905"
## [36] "AU0158_5922" "AU0158_5923" "AU0158_6197"
```

```r
# Plot number of substitutions for each gene with significantly elevated count
par(mar = c(7,4,1,1))
nSubsSignif = matrix(nSubs[which(subRatePVal<0.01)])
rownames(nSubsSignif) = subRateSignif
nSubsSignifSorted = nSubsSignif[order(nSubsSignif[,1]),]
bp = barplot(nSubsSignifSorted, axes = FALSE, axisnames = FALSE)
mtext(side = 2, text = "Number of substitutions", line = 2, cex = 1.5)
axis(2)
axis(1, at = bp, labels = names(nSubsSignifSorted), cex.axis = 1, las = 3)
```

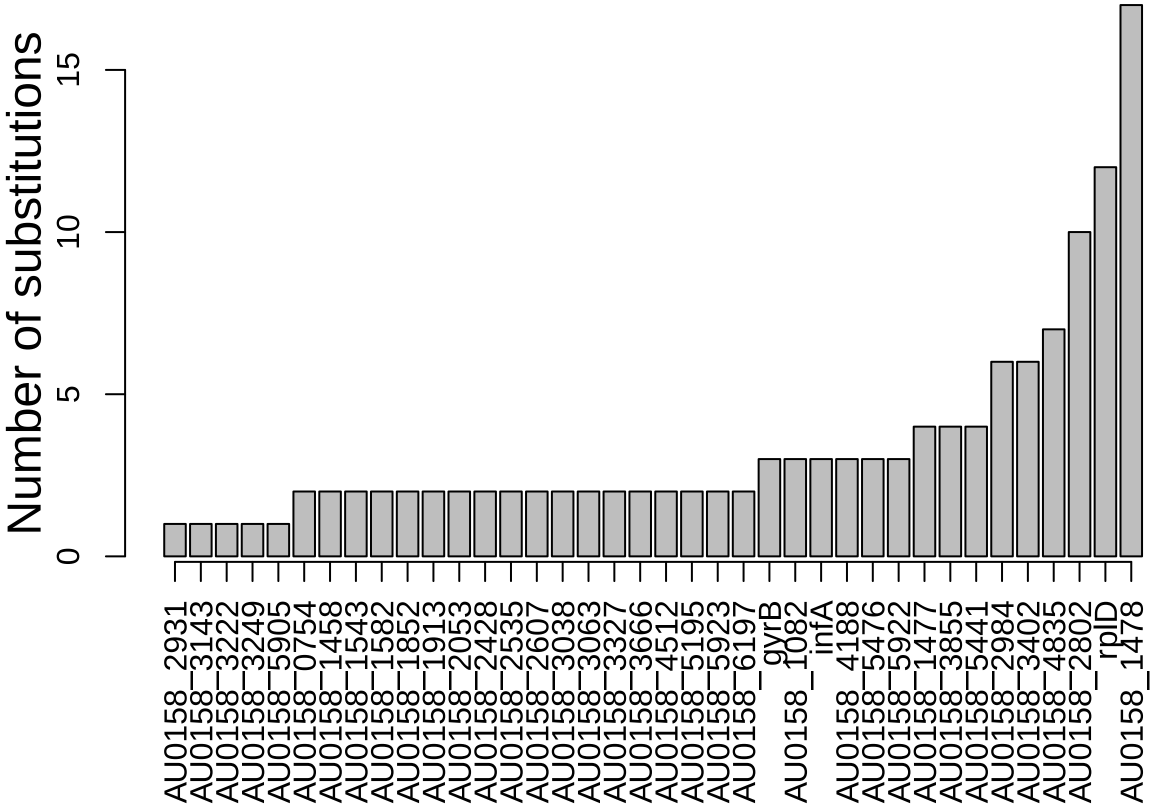

Which of these genes have a significantly different number of substitutions than expected by chance? Of course, some genes may be enriched for substitutions across the tree because they have an intrinsically higher mutation rate and may misleadingly imply that positive selection has acted on this gene. In this case, numbers of non-synonymous and synonymous substitutions are expected to increase by the same amount. For this reason, the next approach tests for a significantly different number of non-synonymous substitutions (upon which selection can act) than synonymous substitutions (which are assumed to evolve neutrally) in order to identify those genes under selection.

B) Estimates of $\mathrm{d_N/d_S}$

Due to the structure of the genetic code, random mutations generate a greater number of non-synonymous than synonymous substitutions. Therefore, in order to estimate $\mathrm{d_N/d_S}$, the ratio of raw counts of non-synonymous and synonymous substitutions have to be adjusted by the ratio that one would expect to see in the absence of any selection (i.e. under neutrality). The null hypothesis (H_0) is that the ratio of non-synonymous and synonymous counts does not significantly differ from the ratio expected by chance (r_0).

First, it is necessary to calculate the ratio expected under neutrality, r_0. This requires calculation of the codon substitution rate matrix, which describes the probability of substituting from one codon to another. The Nielsen and Yang (NY98) model of codon substitution is similar to the HKY85 model of nucleotide substitution and also includes a parameter ω, which represents the value of $\mathrm{d_N/d_S}$.

We have already estimated κ in the PhyML analysis and calculated the empirical codon frequencies in Step 3. We also know that $\mathrm{d_N/d_S} = 1$ under H_0. Therefore, we can estimate the expected ratio r_0 in R using these values.

```
# Load matrix defining types of substitutions
chTypes = read.table("inputData/Codon_change_types.txt",h=T,sep="\t")
# Remove stop codons
```

```r
notStop = (chTypes[3:66,1])!="STOP"
ct61 = matrix(as.integer(as.matrix(chTypes[3:66,4:67][notStop,notStop])),nrow=61)
ct61[lower.tri(ct61)] = t(ct61)[lower.tri(ct61)]
# Calculate the NY98 substitution rate matrix
makeQ = function(kappa,omega,pi=rep(1/61,61),chTypes) {
  aa = chTypes[3:66,1]
  notStop = aa!="STOP"
  C = matrix(as.integer(as.matrix(chTypes[3:66,4:67][notStop,notStop])),nrow=61)
  Q = matrix(0,nrow=61,ncol=61)
  Q[C==2] = 1
  Q[C==3] = kappa
  Q[C==4] = omega
  Q[C==5] = kappa*omega
  Q[lower.tri(Q)] = t(Q)[lower.tri(Q)]
  Q = t(Q * pi)
  diag(Q) = -apply(Q,1,sum)
  Q = Q/sum(-pi*diag(Q))
  return(Q)
}
kappa = 2.222 # Estimate during PhyML analysis in Step 2
dndsNull = 1.0 # Under the null hypothesis
codonCount = as.vector(codonUsage[unlist(geneticCode)!="STO"]) # Exclude stop codons
codonFreq = codonCount/sum(codonCount) # Normalize
# Relative frequency of each substitution
ny98 = makeQ(kappa,dndsNull,codonFreq,chTypes)*codonFreq
# Define the expected rate of various types of substitutions
dsTv = sum(ny98[!is.na(ct61) & ct61==2]) # Synonymous transversions
dsTs = sum(ny98[!is.na(ct61) & ct61==3]) # Synonymous transitions
dnTv = sum(ny98[!is.na(ct61) & ct61==4]) # Non-synonymous transversions
dnTs = sum(ny98[!is.na(ct61) & ct61==5]) # Non-synonymous transitions
# Compute the relative frequency of non-synonymous vs synonymous
# Substitutions under the null hypothesis
(r0 = (dnTv+dnTs)/(dsTv+dsTs))
```

```
## [1] 5.004439
```

```r
# Convert r0 to a frequency
(f0 = r0/(1+r0))
```

```
## [1] 0.8334565
```

This shows that under H_0, we expect to see many more non-synonymous than synonymous substitutions. Since many of the counts of non-synonymous and synonymous substitution for each gene are zero, there is not sufficient power to estimate d_N/d_S for each gene. Therefore, we will group genes according to the number of times they are independently mutated (which was calculated in Step 5A) and estimate d_N/d_S per group.

```r
subsPerGene = cbind(rawNS, nSubs)
# Print the number of genes in each substitution group
table(t(subsPerGene[,3]))
```

```
##
##    0    1    2    3    4    6    7   10   12   17
## 6280  283   23    7    3    2    1    1    1    1
```

```
# Group genes by the total number of substitutions
aggCountsSubs = aggregate(subsPerGene, list(subsPerGene$nSubs), sum)
head(aggCountsSubs)
```

```
##   Group.1   N   S nSubs
## 1       0   0   0     0
## 2       1 181 102   283
## 3       2  37   9    46
## 4       3  21   0    21
## 5       4  10   2    12
## 6       6  12   0    12
```

The table "aggCountsSubs" shows that there are only two synonymous substitutions in genes mutated more than three times. Therefore, we will pool these counts into a single category to estimate d_N/d_S for genes mutated 3 or more times. We also need to remove the first row from this table, which displays the number of invariant genes.

```
# Select counts for genes mutated one, twice and more than three times
aggCountsSubs2 = rbind(aggCountsSubs[2:3,],
                       colSums(aggCountsSubs[4:nrow(aggCountsSubs),]))
rownames(aggCountsSubs2) = c("1sub", "2subs", ">=3subs")
# Calculate dN/dS for each category
dndsVector = apply(aggCountsSubs2, 1, function(x){
  raw_NS = x[2]/x[3]
  dnds = raw_NS/r0
  return(dnds)
})
# Plot results
bp = barplot(dndsVector, axes = FALSE, axisnames = FALSE)
mtext(side = 2, text = expression('d'[N]~'/d'[S]), line = 2, cex = 1.5)
axis(2)
axis(1, at = bp, cex.axis = 1, las = 1, padj = 1,
     labels = c("Genes mutated\nonce", "Genes mutated\ntwice",
                "Genes mutated\n>= 3 times"))
# Add a red horizontal line to represent the value for dN/dS under neutrality
abline(h = 1, col = "red")
```

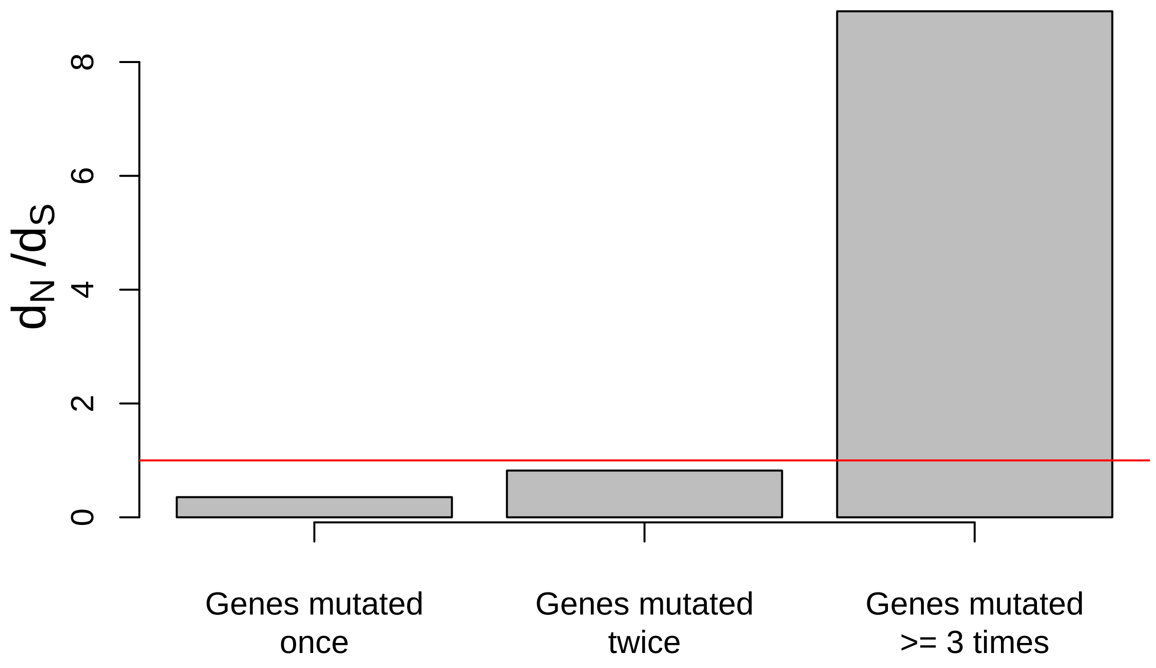

We need to perform a statistical test to see whether the estimate of d_N/d_S for each substitution category is significantly different from r_0. Use a binomial test for each gene to get a p-value and then rank the genes by p-value.

```
dndsPVal = apply(aggCountsSubs2,1,function(x){
  if((x[1]+x[2])==0){
    return(1)
  }
  t = binom.test(x[1],x[1]+x[2],p=f0)
  t$p.value
})
# Print out p-value for dN/dS estimate for each substitution category
(dndsPVal)
```

```
##          1sub         2subs       >=3subs
## 1.896352e-139  8.178442e-27  1.434346e-32
```

Do the values of d_N/d_S for any substitution categories differ significantly from that expected under neutrality? If so, are these genes under positive or purifying selection? It seems that there's some support for the hypothesis that genes with a greater number of substitutions are under stronger positive selection.

We might also want to test the hypothesis that the d_N/d_S across all genes is significantly different from the null hypothesis ($d_N/d_S = 1$). This is achieved by summing all counts of non-synonymous and synonymous substitutions across genes.

```
# Raw N/S across all genes
rawNSCombined = sum(rawNS[,1])/sum(rawNS[,2])
# dNdS estimate across all genes
dndsCombined = (sum(rawNS[,1])/sum(rawNS[,2]))/r0
(grandNullTest = binom.test(sum(rawNS[,1]),sum(rawNS),p=f0))
```

```
##
```

```
##  Exact binomial test
##
## data:  sum(rawNS[, 1]) and sum(rawNS)
## number of successes = 307, number of trials = 420, p-value =
## 1.261e-07
## alternative hypothesis: true probability of success is not equal to 0.8334565
## 95 percent confidence interval:
##  0.6858189 0.7727976
## sample estimates:
## probability of success
##              0.7309524
```

```
# Significance of raw N/S across all genes
grandNullTest$p.value/(1-grandNullTest$p.value)
```

```
## [1] 1.261383e-07
```

```
# Significance of dN/dS across all genes
grandNullTest$p.value/(1-grandNullTest$p.value)/r0
```

```
## [1] 2.520528e-08
```

What does the magnitude of the p-value suggest about the power to detect patterns at the whole-genome versus groups of genes? In estimating a genome-wide average of d_N/d_S, it is possible that regions of the genome where d_N/d_S is significantly greater than r_0 will be missed.

C) The McDonald-Kreitman test

The McDonald-Kreitman (MK) test compares the number of non-synonymous to synonymous substitutions that are fixed between groups (d_N/d_S) with those that occur as polymorphisms within groups (P_N/P_S). Here, each group is a population of isolates sampled from the same patient. The first step is to identify which substitutions occur within a population (i.e. substitutions that occur in samples from a single patient).

```
# Get patient identifier from sequence label (first letter)
patientId = sapply(newSeqLabels,substr,1,1)
# Number of patients
nPatients = nlevels(factor(patientId))
# For each variable site, count check whether a snp is unique to one patient
uniqueSnp = apply(vSites,2,function(x){
  sum(rowSums(table(x,patientId)==0)!=(nPatients-1))==1
  })
table(uniqueSnp)
```

```
## uniqueSnp
## FALSE  TRUE
##   105   406
```

```
# Aggregate counts according to whether the substitution is unique to the patient
aggUniqueSnps = aggregate(subAnnotation[,6:7], list(uniqueSnp), sum)
head(aggUniqueSnps)
```

```
##   Group.1 NonSynCount SynCount
## 1   FALSE          80       28
## 2    TRUE         227       85
```

Now we have a contingency table of counts, we can estimate the odds ratio $(D_{\mathrm{N}}\ P_{\mathrm{S}})/(D_{\mathrm{S}}\ P_{\mathrm{N}})$ and carry out a Fisher's exact test and a Chi Square test to find out whether the differences in counts between and within patients is significant. This tests for an association between the groups (between and within patients) and the outcome (the count of each substitution type).

```
oddsRatio = ((aggUniqueSnps$NonSynCount[1] * aggUniqueSnps$SynCount[2])/
               (aggUniqueSnps$SynCount[1] * aggUniqueSnps$NonSynCount[2]))
# Print odds ratio
(oddsRatio)
```

```
## [1] 1.069855
```

```
fisher.test(aggUniqueSnps)
```

```
##
##  Fisher's Exact Test for Count Data
##
## data:  aggUniqueSnps
## p-value = 0.9256
## alternative hypothesis: two.sided
```

```
chisq.test(aggUniqueSnps)
```

```
##
##  Pearson's Chi-squared test
##
## data:  aggUniqueSnps
## X-squared = 0.41681, df = 2, p-value = 0.8119
```

The MK test shows that there is no significant different between $D_{\mathrm{N}}/D_{\mathrm{S}}$ and $P_{\mathrm{N}}/P_{\mathrm{S}}$, implying that the population is evolving neutrally at the genome level.

Summary

This exercise has analyzed the genome sequences of 112 *B. dolosa* sampled longitudinally from multiple, chronically infected patients. We have visualized the evolutionary history of this population by constructing a phylogenetic tree, which showed clustering by both patient and sampling time. The estimate of the evolutionary rate is consistent with the published estimate for this species (1) and other short-term evolutionary rates in human bacterial pathogens (5,6,7). By carrying out genome annotation and ancestral sequence reconstruction, we have also been able to investigate the selective pressures acting on the population. We found that genes mutated more than three times across the phylogeny are under strong positive selection. These include *rplD* (an rRNA binding protein involved in conferring macrolide resistance), *gyrA* (involved in conferring quinolone resistance) and *infA* (translation initiation factor).

References

1. Lieberman TD, Michel J-B, Aingaran M, Potter-Bynoe G, Roux D, Davis MR, et al. Parallel bacterial evolution within multiple patients identifies candidate pathogenicity genes. Nat Genet. 2011;43(12):1275–80
2. Guindon S, Gascuel O. A simple, fast, and accurate algorithm to estimate large phylogenies by maximum likelihood. Syst Biol. 2003;52:696–704.
3. Ashkenazy H, Penn O, Doron-Faigenboim A, Cohen O, Cannarozzi G, Zomer O, et al. FastML: A web server for probabilistic reconstruction of ancestral sequences. Nucleic Acids Res. 2012;40:W580-4
4. Rutherford K, Parkhill J, Crook J, Horsnell T, Rice P, Rajandream MA, et al. Artemis: sequence visualization and annotation. Bioinformatics. 2000;16(10):944–5.
5. Eyre DW, Cule ML, Wilson DJ, Griffiths D, Vaughan A, O'Connor L, et al. Diverse sources of *C. difficile* infection identified on whole-genome sequencing. N Engl J Med. 2013;369(13):1195–205.
6. Ford CB, Lin PL, Chase MR, Shah RR, Iartchouk O, Galagan J, et al. Use of whole genome sequencing to estimate the mutation rate of *Mycobacterium tuberculosis* during latent infection. Nat Genet. 2011;43(5):482–6.
7. Morelli G, Didelot X, Kusecek B, Schwarz S, Bahlawane C, Falush D, et al. Microevolution of *Helicobacter pylori* during prolonged infection of single hosts and within families. PLoS Genet. 2010;6(7):e1001036.
