# Supplementary material for: Practical Approaches for Detecting Selection in Microbial Genomes
Source: PLoS Comput Biol. 2016 Feb 11;12(2):e1004739. doi: 10.1371/journal.pcbi.1004739 (PMC4750996; doi:10.1371/journal.pcbi.1004739)
Supplement: S1 Table — A selection of published analyses employing the methods described in Steps 1–5 to address a range of evolutionary questions across different microbial species. (PDF) [file pcbi.1004739.s002.pdf]

Study	Research question	Phylogenetic tree construction	Evolutionary rate estimation	Genome annotation	Selection detection	Additional comments
He et al. 2010 (1)	How does *Clostridium difficile* adapt over different evolutionary scales?	Maximum likelihood analysis for the full data (RAxML (2)) and a subset of virulent isolates (PhyML (3))	Bayesian MCMC analysis (BEAST)	Artemis (4)	Per-gene d_N/d_S estimates	Recombination detection (ClonalFrame)
Lieberman and Michel et al. 2011 (5)	How does *Burkholderia dolosa* adapt within patients over time?	Maximum likelihood analysis (DNAML (PHYLIP) (6))	Linear regression	Manual annotation of genes under positive selection	d_N/d_S estimation for genes independently mutated once, twice or at least three times	Genome-wide association study to test for SNPs associated with drug resistance and virulence
Mutreja et al. 2011 (7)	What are the evolutionary dynamics of *Vibrio cholera* pandemics?	Maximum likelihood analysis in RAxML.	Linear regression (Path-O-Gen (8)) and Bayesian MCMC analysis (BEAST)	NA	NA	Recombination detection using method by (9) (similar to Gubbins(10))
Lie et al. 2012 (11)	How does the host immune system respond to *Streptococcus pneumoniae* infection?	NA	NA	Glimmer3 (12)	Per-gene d_N/d_S estimates, d_N/d_S estimation per codon using method (13)	
Fahrat et al. 2013 (14)	Which sites in *Mycobacterium tuberculosis* are under positive selection?	Maximum parsimony, maximum likelihood (PhyML) and Bayesian analysis (MrBayes (15))	NA	NA	Per-gene d_N/d_S estimates (PAML), test for elevated density of substitutions in genes and convergent evolution across the tree	
Pepperell et al. 2013 (16)	How does natural selection act on *Mycobacterium tuberculosis* species?	Baysian analysis (BEAST)	Bayesian MCMC analysis (BEAST)	Kodon v 3.62 (17)	McDonald-Kreitman Test (18), relative rates of d_N/d_S estimates per site (PAML)	Recombination detection (SplitsTree (19))
Cornejo et al. 2013 (20)	How has selection acted on the genome of *Streptococcus mutans*?	Maximum likelihood analysis	Use published estimate	NCBI Prokaryotic Genomes Automatic Annotation Pipeline (21)	Analysis of the site frequency spectrum (PrFreq (22)), extension of the McDonald-Kreitman Test for each gene (SnIPRE (23))	Recombination detection (GeneConv (24)), estimation of the relative contribution of recombination to mutation (ClonalFrame)
Golubchik et al. 2014 (25)	How does *Staphylococcus aureus* evolve during asymptomatic carriage?	Maximum likehood analysis for within host data, Bayesian analysis for between host analysis, accounting for recombination (ClonalFrame (26))	Use published estimate	xBASE (27)	Test for elevated substitution rates, McDonald-Kreitman Test	Recombination detection (omegaMap (13))
Grad et al. 2014 (28)	What are the transmission dynamics of a *Neisseria gonorrhoeae* epidemic?	Maximum likelihood analysis (RAxML)	Linear regression (Path-O-Gen) and Bayesian MCMC analysis (BEAST)	NA	NA	
Marvig et al. 2014 (29)	What mutations allow *Pseudomonas aeruginosa* adapt in human hosts?	Maximum parsimony phylogenetic tree construction (PAUP (30))	Use published estimate	NA	Test for convergent evolution across the tree	
Paterson et al. 2015 (31)	How much bacterial genetic diversity is transmitted between individuals during an MRSA outbreak?	Maximum likelihood (RAxML) and Bayesian analysis (MrBayes)	Linear regression (Path-O-Gen) and Bayesian MCMC analysis (BEAST)	NA	NA	

References

1. He M, Sebaihia M, Lawley TD, Stabler RA, Dawson LF, Martin MJ, et al. Evolutionary dynamics of Clostridium difficile over short and long time scales. Proc Natl Acad Sci U S A. 2010;107(16):7527–32.
2. Stamatakis A. RAxML version 8: A tool for phylogenetic analysis and post-analysis of large phylogenies. Bioinformatics. 2014;30(9):1312–3.
3. Guindon S, Gascuel O. A simple, fast, and accurate algorithm to estimate large phylogenies by maximum likelihood. Syst Biol. 2003;52(5):696–704.
4. Rutherford K, Parkhill J, Crook J, Horsnell T, Rice P, Rajandream MA, et al. Artemis: sequence visualization and annotation. Bioinformatics. 2000;16(10):944–5.
5. Lieberman TD, Michel J-B, Aingaran M, Potter-Bynoe G, Roux D, Davis MR, et al. Parallel bacterial evolution within multiple patients identifies candidate pathogenicity genes. Nat Genet. 2011;43(12):1275–80.
6. Felsenstein J. Phylip: phylogeny inference package (version 3.2). Cladistics [Internet]. 1989;5:164–6. Available from: http://evolution.genetics.washington.edu/phylip/faq.html#citation
7. Mutreja A, Kim DW, Thomson NR, Connor TR, Lee JH, Kariuki S, et al. Evidence for several waves of global transmission in the seventh cholera pandemic. Nature. 2011;477(7365):462–5.
8. Rambaut A. Path-O-Gen, v1.4 [Internet]. Available from: http://tree.bio.ed.ac.uk/software/pathogen
9. Croucher NJ, Harris SR, Fraser C, Quail MA, Burton J, van der Linden M, et al. Rapid pneumococcal evolution in response to clinical interventions. Science. 2011;331(6016):430–4.
10. Croucher NJ, Page AJ, Connor TR, Delaney AJ, Keane JA, Bentley SD, et al. Rapid phylogenetic analysis of large samples of recombinant bacterial whole genome sequences using Gubbins. Nucleic Acids Res. 2014;43(3):e15.
11. Li Y, Gierahn T, Thompson CM, Trzciński K, Ford CB, Croucher N, et al. Distinct Effects on Diversifying Selection by Two Mechanisms of Immunity against Streptococcus pneumoniae. PLoS Pathog. 2012;8(11):1002989.
12. Delcher AL, Bratke KA, Powers EC, Salzberg SL. Identifying bacterial genes and endosymbiont DNA with Glimmer. Bioinformatics. 2007;23(6):673–9.
13. Wilson DJ, McVean G. Estimating diversifying selection and functional constraint in the presence of recombination. Genetics. 2006;172(3):1411–25.
14. Farhat MR, Shapiro BJ, Kieser KJ, Sultana R, Jacobson KR, Victor TC, et al. Genomic analysis identifies targets of convergent positive selection in drug-resistant Mycobacterium tuberculosis. Nat Genet. 2013;45(10):1183–9.

15. Ronquist F, Teslenko M, Van Der Mark P, Ayres DL, Darling A, Höhna S, et al. Mrbayes 3.2: Efficient bayesian phylogenetic inference and model choice across a large model space. Syst Biol. 2012;61(3):539–42.

16. Pepperell CS, Casto AM, Kitchen A, Granka JM, Cornejo OE, Holmes EC, et al. The role of selection in shaping diversity of natural M. tuberculosis populations. PLOS Pathog. 2013;9(8):e1003543.

17. Applied Maths. Kodon [Internet]. Available from: http://www.applied-maths.com

18. McDonald JH, Kreitman M. Adaptive protein evolution at the Adh locus in Drosophila. Nature. 1991;351(6328):652–4.

19. Huson DH, Bryant D. Application of phylogenetic networks in evolutionary studies. Mol Biol Evol. 2006;23(2):254–67.

20. Cornejo OE, Lefébure T, Pavinski Bitar PD, Lang P, Richards VP, Eilertson K, et al. Evolutionary and population genomics of the cavity causing bacteria streptococcus mutans. Mol Biol Evol. 2013;30(4):881–93.

21. Tatusova T, DiCuccio M, Badretdin A, Chetvernin V, Ciufo S, Li W. Prokaryotic genome annotation pipeline. In: The NCBI Handbook. 2nd ed. Bethesda (MD): National Center for Biotechnology Information (US); 2013.

22. Boyko AR, Williamson SH, Indap AR, Degenhardt JD, Hernandez RD, Lohmueller KE, et al. Assessing the evolutionary impact of amino acid mutations in the human genome. PLoS Genet. 2008;4(5):e1000083.

23. Eilertson KE, Booth JG, Bustamante CD. SnIPRE: Selection Inference Using a Poisson Random Effects Model. PLoS Comput Biol. 2012;8(12):e1002806.

24. Sawyer S. Statistical tests for detecting gene conversion. Mol Biol Evol. 1989;6(5):526–38.

25. Golubchik T, Batty EM, Miller RR, Farr H, Young BC, Larner-Svensson H, et al. Within-Host Evolution of Staphylococcus aureus during Asymptomatic Carriage. PLoS One. 2013;8(5):e61319.

26. Didelot X, Falush D. Inference of bacterial microevolution using multilocus sequence data. Genetics. 2007;175(3):1251–66.

27. Chaudhuri RR, Pallen MJ. xBASE, a collection of online databases for bacterial comparative genomics. Nucleic Acids Res. 2006;34:D335–7.

28. Grad YH, Kirkcaldy RD, Trees D, Dordel J, Harris SR, Goldstein E, et al. Genomic epidemiology of Neisseria gonorrhoeae with reduced susceptibility to cefixime in the USA: A retrospective observational study. Lancet Infect Dis. 2014;14(3):220–6.

29. Marvig RL, Sommer LM, Molin S, Johansen HK. Convergent evolution and adaptation of Pseudomonas aeruginosa within patients with cystic fibrosis. Nat Genet. 2014;47(1):57–64.

30. Swofford DL. PAUP* phylogenetic analysis using parsimony (*and other methods). Version 4. Sunderland, Massachusetts, USA: Sinauer Associates; 2003.

31. Paterson GK, Harrison EM, Murray GGR, Welch JJ, Warland JH, Holden MTG, et al. Capturing the cloud of diversity reveals complexity and heterogeneity of MRSA carriage, infection and transmission. Nat Commun. 2015;6:6560.
